# Supplementary material for: Stilbenoids and Flavonoids from Cajanus cajan (L.) Millsp. and Their α-Glucosidase Inhibitory Activities
Source: Molecules. 2023 Apr 27;28(9):3779. doi: 10.3390/molecules28093779 (PMC10180137; doi:10.3390/molecules28093779)
Supplement: Supplementary file 1 [file molecules-28-03779-s001.zip › molecules-2341995-supplementary.pdf]

Stilbenoids and Flavonoids from *Cajanus cajan* (L.) Millsp. and Their *α*-Glucosidase Inhibitory Activities

Yaxian Zhao [1,2,3], Xinman Zhao [1,2,3], Mengjia Guo [1,2,3], Krishnapriya M. Varier [1,2,3], Babu Gajendran [1,2,3],
Shaohuan Liu [1,2,3], Ling Tao [1,2,3], Xiangchun Shen [1,2,3,*] and Nenling Zhang [1,2,3,*]

[1] The State Key Laboratory of Functions and Applications of Medicinal Plants, School of Pharmaceutical Sciences, Guizhou Medical University, Guiyang 550025, China; zhaoyaxian@stu.gmc.edu.cn (Y.Z.)
[2] The High Efficacy Application of Natural Medicinal Resources Engineering Center of Guizhou Province, School of Pharmaceutical Sciences, Guizhou Medical University, Guiyang 550025, China
[3] The Key Laboratory of Optimal Utilization of Natural Medicine Resources, School of Pharmaceutical Sciences, Guizhou Medical University, Guiyang 550025, China
* Correspondence: shenxiangchun@126.com (X.S.); zhangnenling@gmc.edu.cn (N.Z.)

Two new stilbenoids, cajanstilbenoid C (**1**) and cajanstilbenoid D (**2**), together with eight other known stilbenoids (**3-10**) and seventeen known flavonoids (**11-27**) were isolated from the petroleum ether and ethyl acetate portions of the 95% ethanol extract of leaves of *Cajanus cajan* (L.) Millsp.. The planar structures of the new compounds were elucidated by NMR, high-resolution mass spectrometry, and their absolute configurations were determined by comparison of their experimental and calculated electronic circular dichroism (ECD) values. All the compounds were assayed for their inhibitory activities against yeast *α*-glucosidase. The results demonstrated that compounds **3**, **8-9**, **11**, **13**, **19-21** and **24-26** had strong inhibitory activities against *α*-glucosidase, with compound **11** (IC_{50} = 0.87 ± 0.05 μM) exhibiting the strongest activity. The structure-activity relationships were preliminarily summarized. Moreover, enzyme kinetics showed that compound **8** was a non-competitive inhibitor, compounds **11**, **24-26** were anti-competitive and compounds **9** and **13**

Citation: Zhao, Y.; Zhao, X.; Guo, M.; Varier, K.M.; Gajendran, B.; Liu, S.; Tao, L.; Shen, X.; Zhang, N. Stilbenoids and Flavonoids from *Cajanus cajan* (L.) Millsp. and Their *α*-Glucosidase Inhibitory Activities. *Molecules* **2023**, *28*, x. https://doi.org/10.3390/xxxxx

Academic Editors: George Grant, Anna Choromańska and Nina Rembiałkowska

Received: 29 March 2023
Revised: 23 April 2023
Accepted: 25 April 2023
Published: date

Copyright: © 2023 by the authors. Submitted for possible open access publication under the terms and conditions of the Creative Commons Attribution (CC BY) license (https://creativecommons.org/licenses/by/4.0/).

were mixed competitive.

Keywords: *Cajanus cajan*; stilbenoid; flavonoid; *α*-glucosidase inhibitory activity

List of supporting information

Figure S1: HR-ESI-MS Spectrum of Compound **1**
Figure S2: UV Spectrum of Compound **1**
Figure S3: IR Spectrum of Compound **1**
Figure S4: ^{1}H-NMR Spectrum of Compound **1**
Figure S5: ^{13}C-NMR Spectrum of Compound **1**
Figure S6: HSQC Spectrum of Compound **1**
Figure S7: HMBC Spectrum of Compound **1**
Figure S8: NOESY Spectrum of Compound **1**
Figure S9: HR-ESI-MS Spectrum of Compound **2**
Figure S10: UV Spectrum of Compound **2**
Figure S11: IR Spectrum of Compound **2**
Figure S12: ^{1}H-NMR Spectrum of Compound **2**
Figure S13: ^{13}C-NMR Spectrum of Compound **2**
Figure S14: HSQC Spectrum of Compound **2**
Figure S15: HMBC Spectrum of Compound **2**
Figure S16: NOESY Spectrum of Compound **2**
Figure S17: ^{1}H-NMR Spectrum of Compound **3**
Figure S18: ^{13}C-NMR Spectrum of Compound **3**
Figure S19: ^{1}H-NMR Spectrum of Compound **4**
Figure S20: ^{13}C-NMR Spectrum of Compound **4**
Figure S21: ^{1}H-NMR Spectrum of Compound **5**
Figure S22: ^{13}C-NMR Spectrum of Compound **5**
Figure S23: ^{1}H-NMR Spectrum of Compound **6**
Figure S24: ^{13}C-NMR Spectrum of Compound **6**
Figure S25: ^{1}H-NMR Spectrum of Compound **7**
Figure S26: ^{13}C-NMR Spectrum of Compound **7**
Figure S27: ^{1}H-NMR Spectrum of Compound **8**
Figure S28: ^{13}C-NMR Spectrum of Compound **8**
Figure S29: ^{1}H-NMR Spectrum of Compound **9**
Figure S30: ^{13}C-NMR Spectrum of Compound **9**
Figure S31: ^{1}H-NMR Spectrum of Compound **10**
Figure S32: ^{13}C-NMR Spectrum of Compound **10**
Figure S33: ^{1}H-NMR Spectrum of Compound **11**
Figure S34: ^{13}C-NMR Spectrum of Compound **11**
Figure S35: ^{1}H-NMR Spectrum of Compound **12**
Figure S36: ^{13}C-NMR Spectrum of Compound **12**
Figure S37: ^{1}H-NMR Spectrum of Compound **13**
Figure S38: ^{1}H-NMR Spectrum of Compound **14**
Figure S39: ^{13}C-NMR Spectrum of Compound **14**
Figure S40: ^{1}H-NMR Spectrum of Compound **15**
Figure S41: ^{13}C-NMR Spectrum of Compound **15**
Figure S42: ^{1}H-NMR Spectrum of Compound **16**
Figure S43: ^{13}C-NMR Spectrum of Compound **16**

Figure S44: ^{1}H-NMR Spectrum of Compound **17**
Figure S45: ^{13}C-NMR Spectrum of Compound **17**
Figure S46: ^{1}H-NMR Spectrum of Compound **18**
Figure S47: ^{13}C-NMR Spectrum of Compound **18**
Figure S48: ^{1}H-NMR Spectrum of Compound **19**
Figure S49: ^{13}C-NMR Spectrum of Compound **19**
Figure S50: ^{1}H-NMR Spectrum of Compound **20**
Figure S51: ^{13}C-NMR Spectrum of Compound **20**
Figure S52: ^{1}H-NMR Spectrum of Compound **21**
Figure S53: ^{13}C-NMR Spectrum of Compound **21**
Figure S54: ^{1}H-NMR Spectrum of Compound **22**
Figure S55: ^{13}C-NMR Spectrum of Compound **22**
Figure S56: ^{1}H-NMR Spectrum of Compound **23**
Figure S57: ^{13}C-NMR Spectrum of Compound **23**
Figure S58: ^{1}H-NMR Spectrum of Compound **24**
Figure S59: ^{13}C-NMR Spectrum of Compound **24**
Figure S60: ^{1}H-NMR Spectrum of Compound **25**
Figure S61: ^{13}C-NMR Spectrum of Compound **25**
Figure S62: ^{1}H-NMR Spectrum of Compound **26**
Figure S63: ^{13}C-NMR Spectrum of Compound **26**
Figure S64: ^{1}H-NMR Spectrum of Compound **27**
Figure S65: ^{13}C-NMR Spectrum of Compound **27**

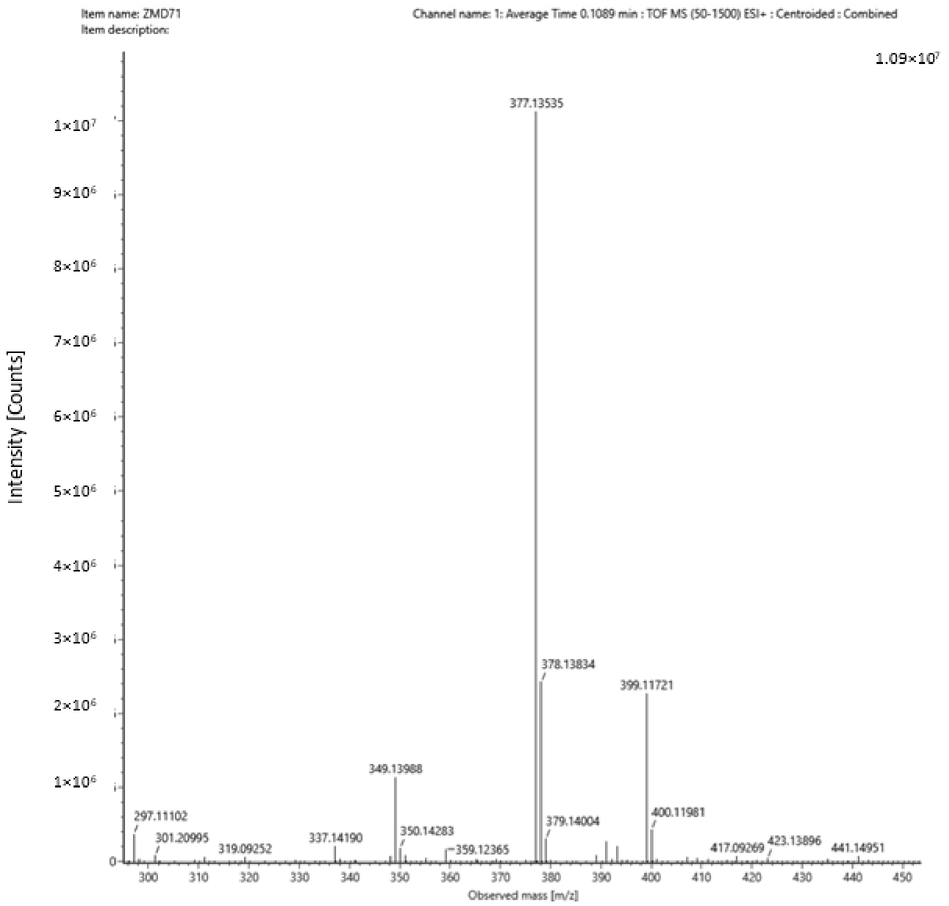

Composition	i-FIT Confidence (%)	Predicted m/z	m/z error (PPM)
C21H22O5Na	99.681634	377.135945	-1.581625

Figure S1: HR-ESI-MS Spectrum of Compound **1**

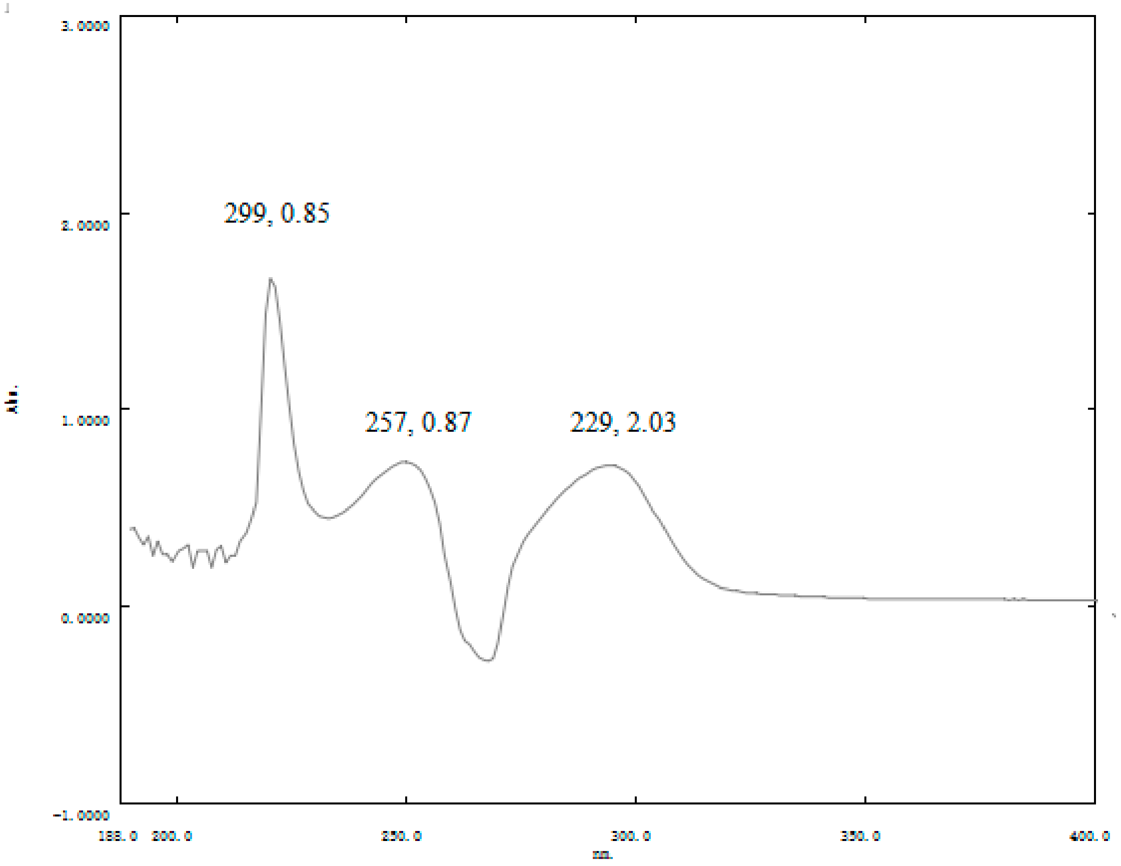

Figure S2: UV Spectrum of Compound **1**

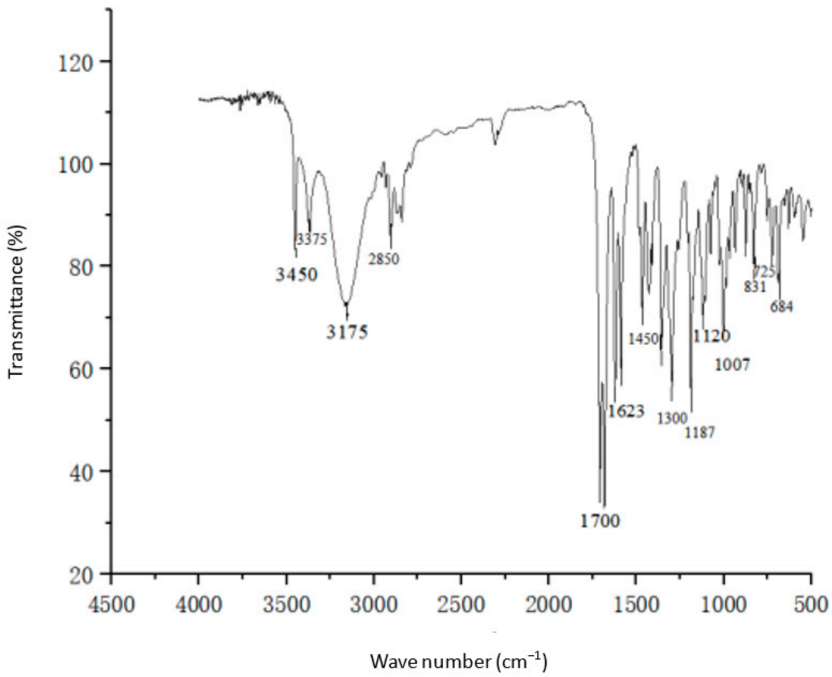

Figure S3: IR Spectrum of Compound **1**

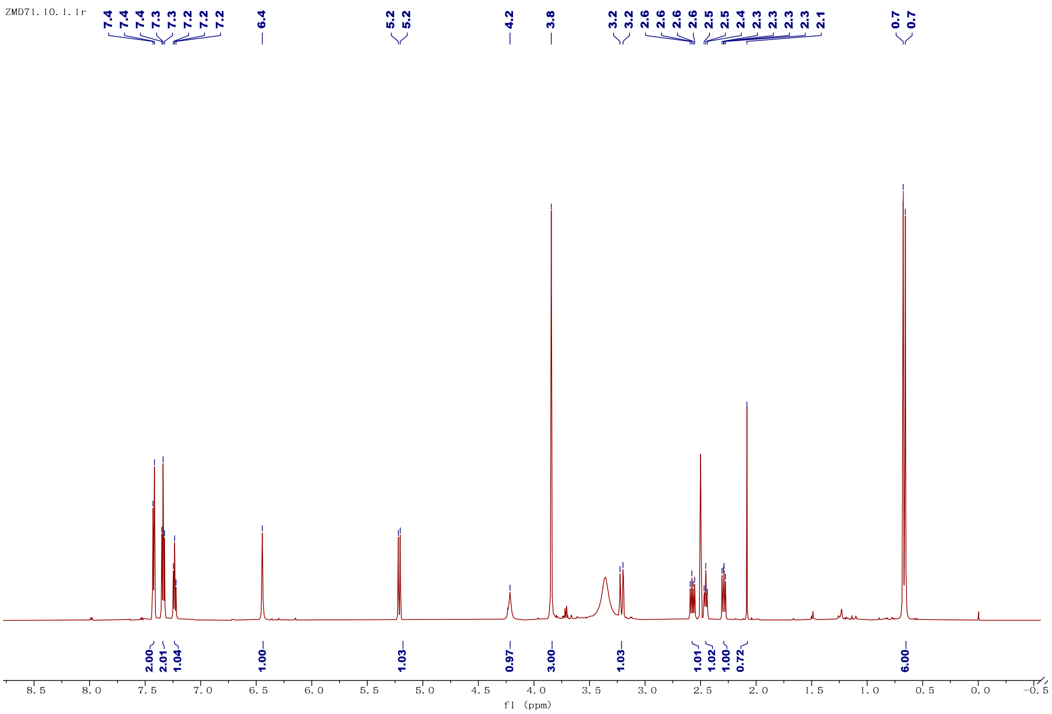

Figure S4: ^{1}H-NMR Spectrum of Compound **1**

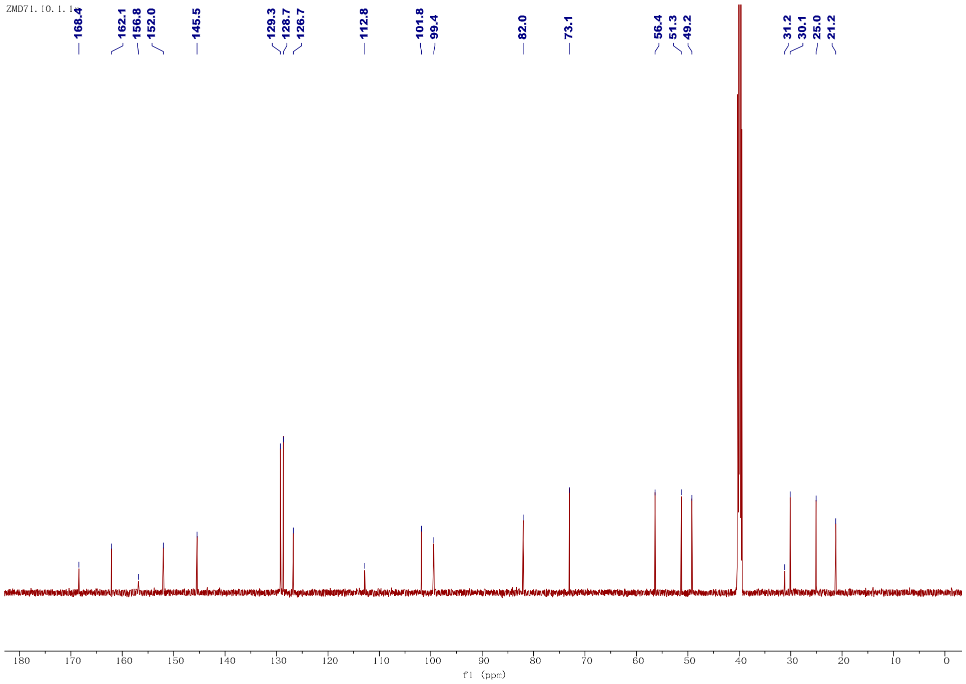

Figure S5: ^{13}C-NMR Spectrum of Compound **1**

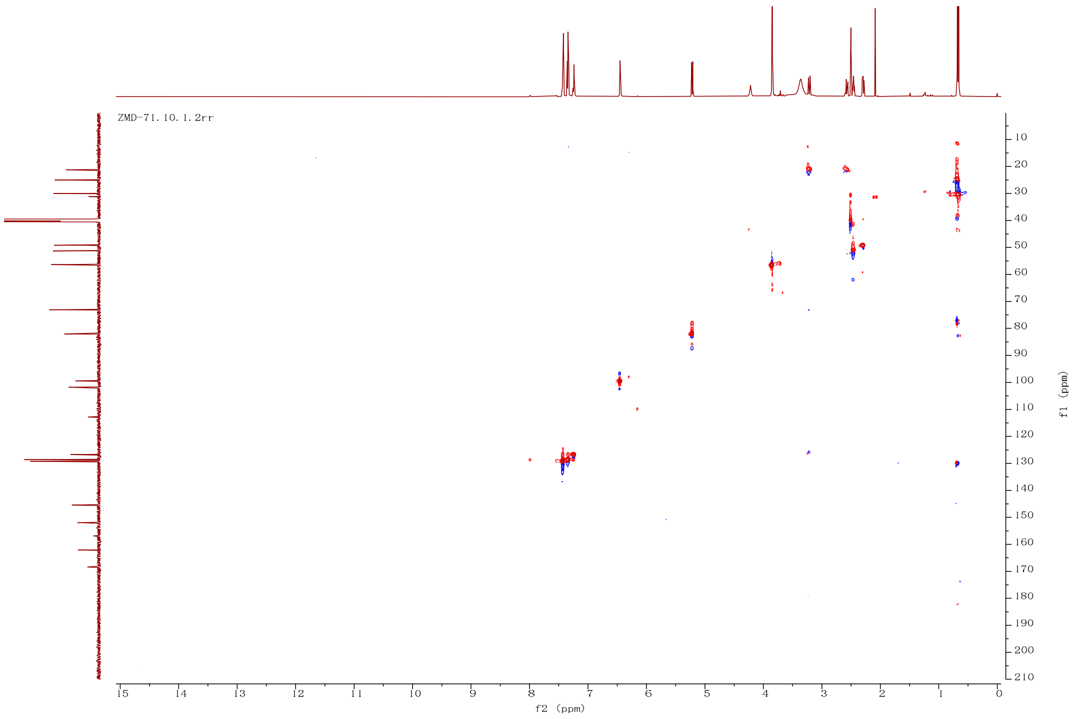

Figure S6: HSQC Spectrum of Compound **1**

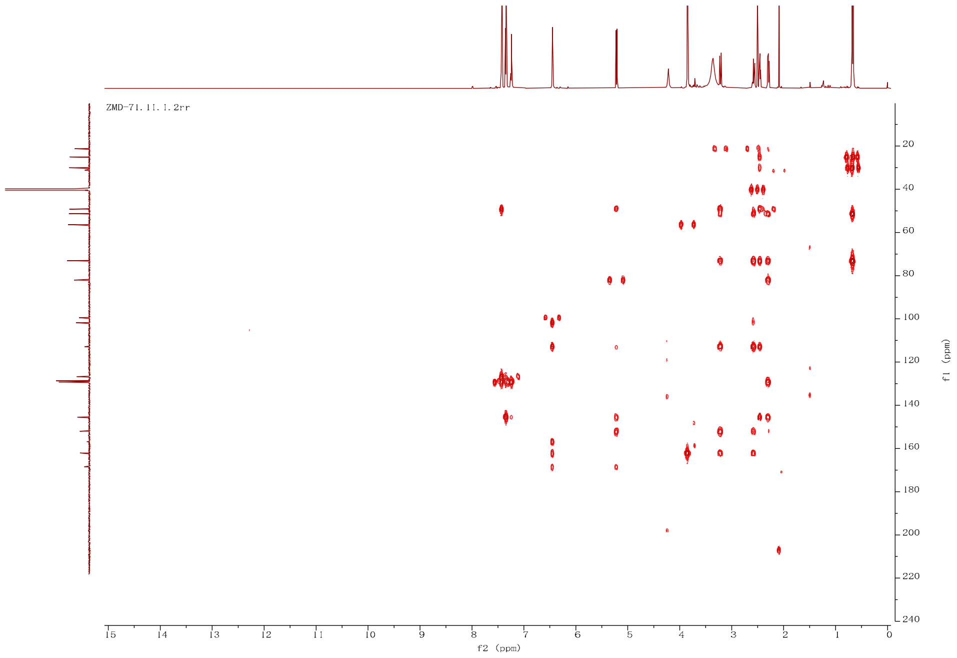

Figure S7: HMBC Spectrum of Compound **1**

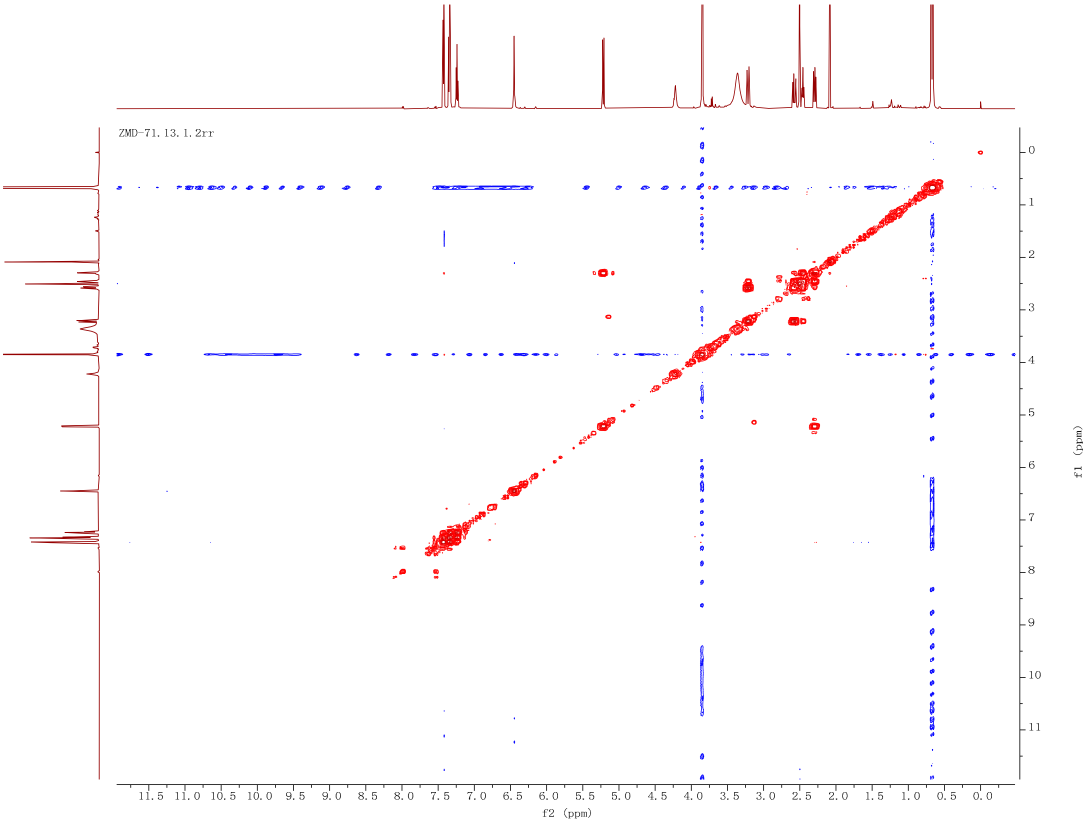

Figure S8: NOESY Spectrum of Compound **1**

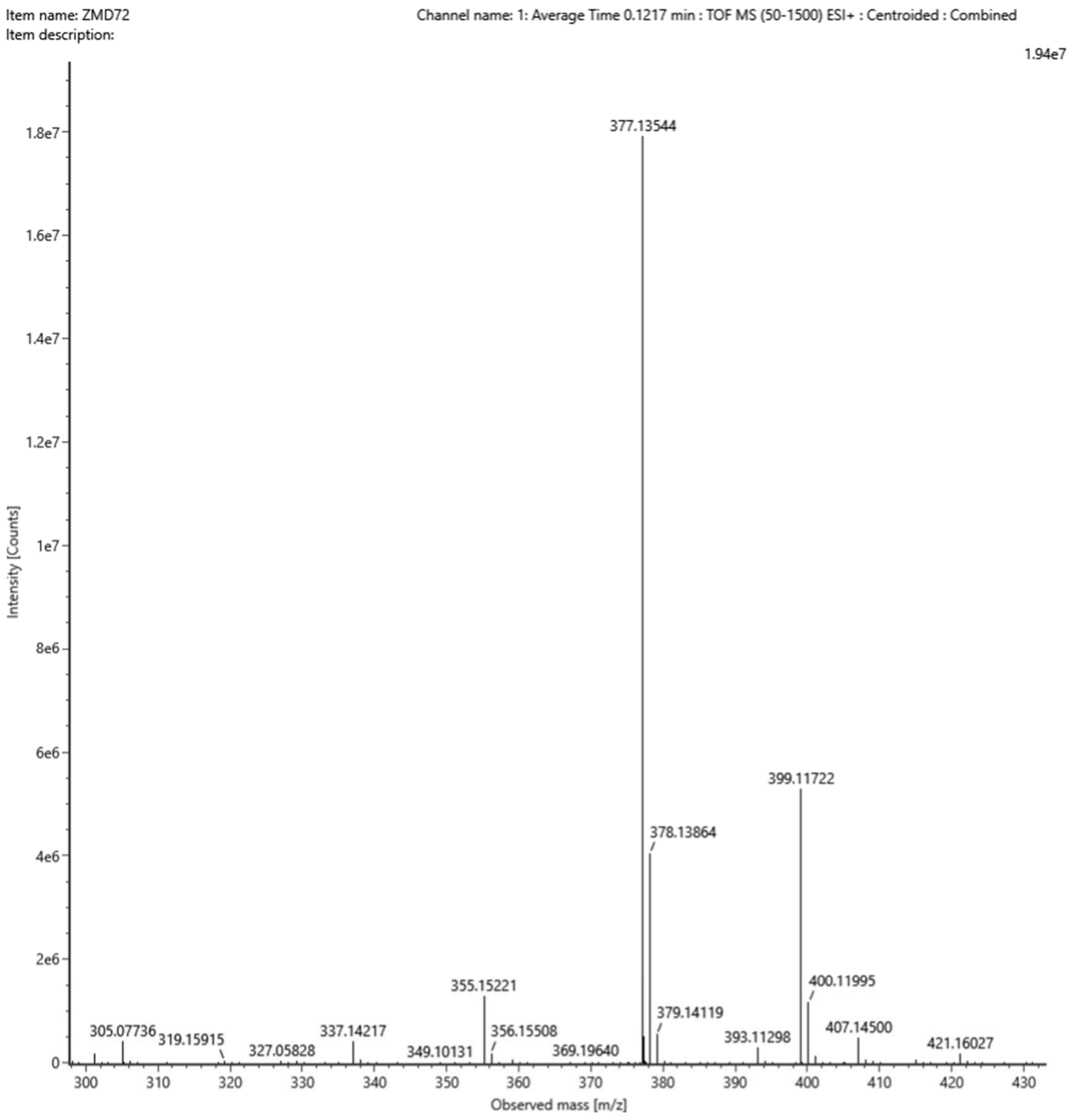

Composition	i-FIT Confidence (%)	Predicted m/z	m/z error (PPM)
C21H22O5Na	99.992012	377.135945	-1.342344

Figure S9: HR-ESI-MS Spectrum of Compound **2**

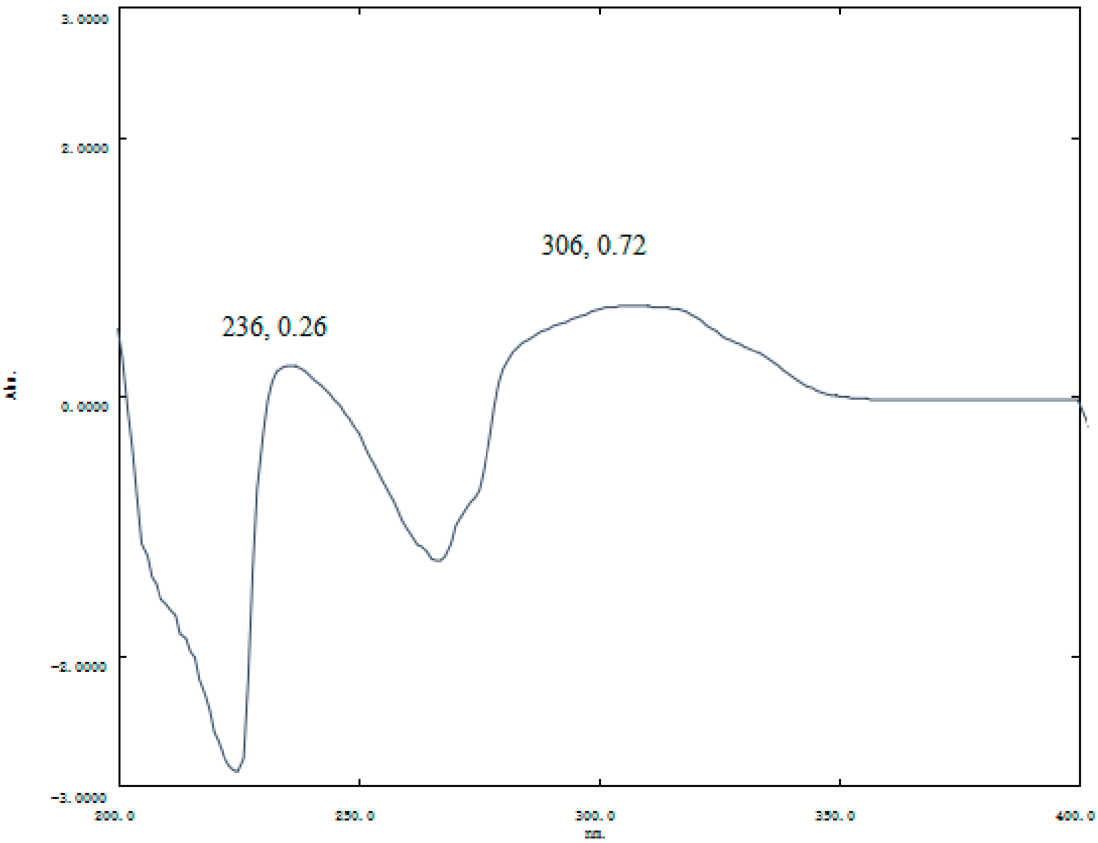

Figure S10: UV Spectrum of Compound **2**

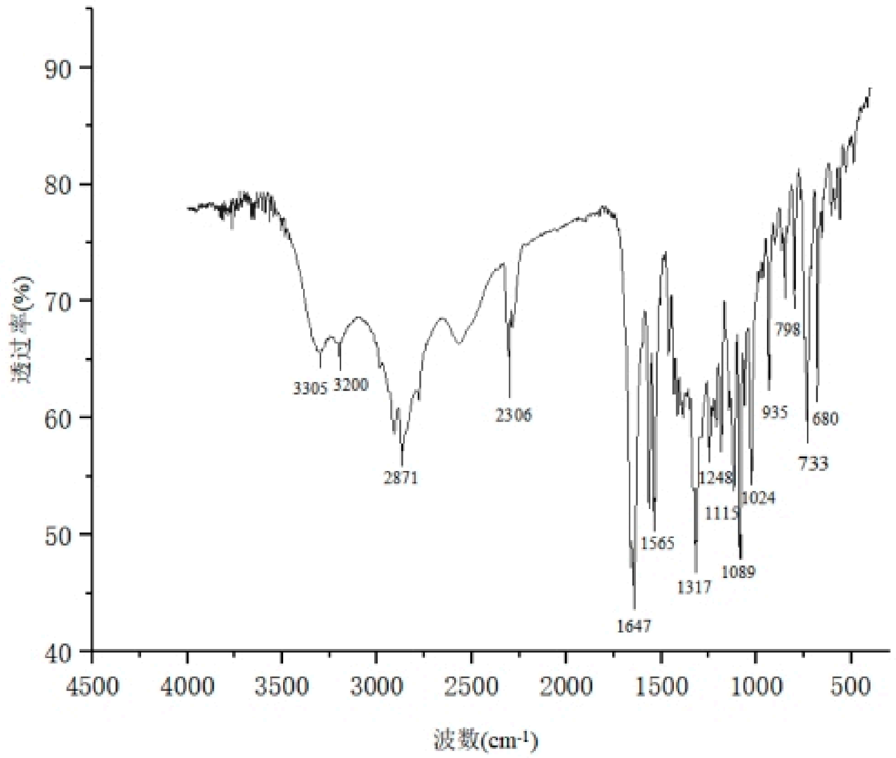

Figure S11: IR Spectrum of Compound **2**

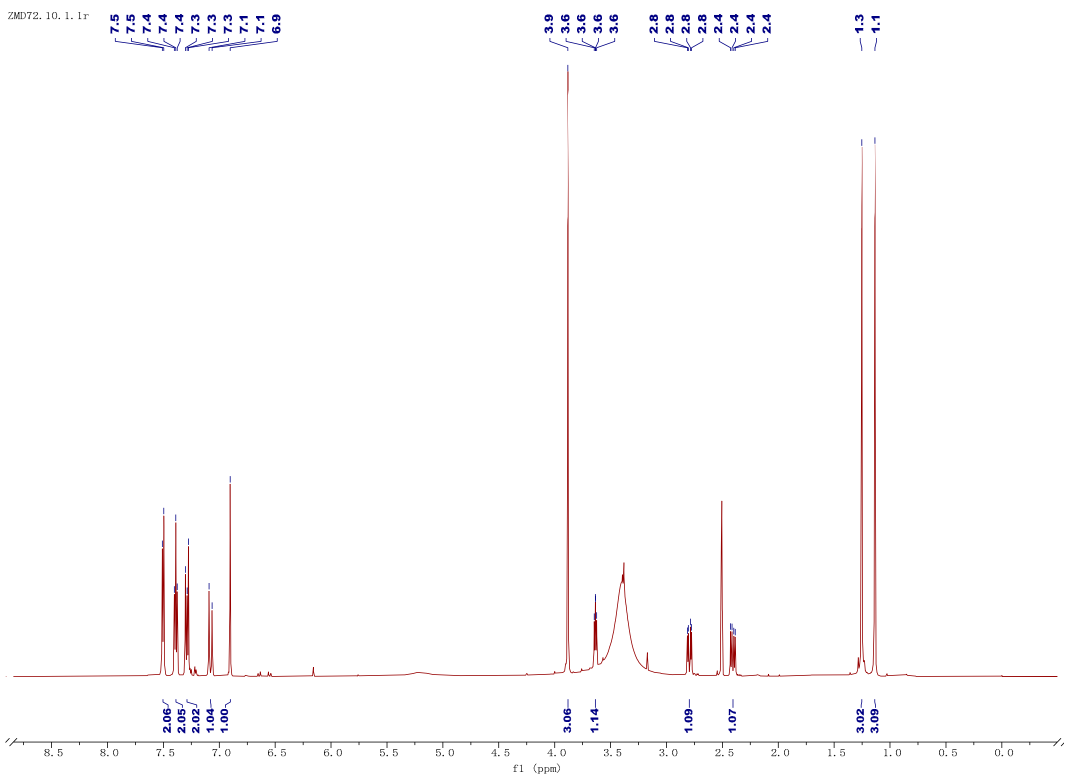

Figure S12: ^{1}H-NMR Spectrum of Compound **2**

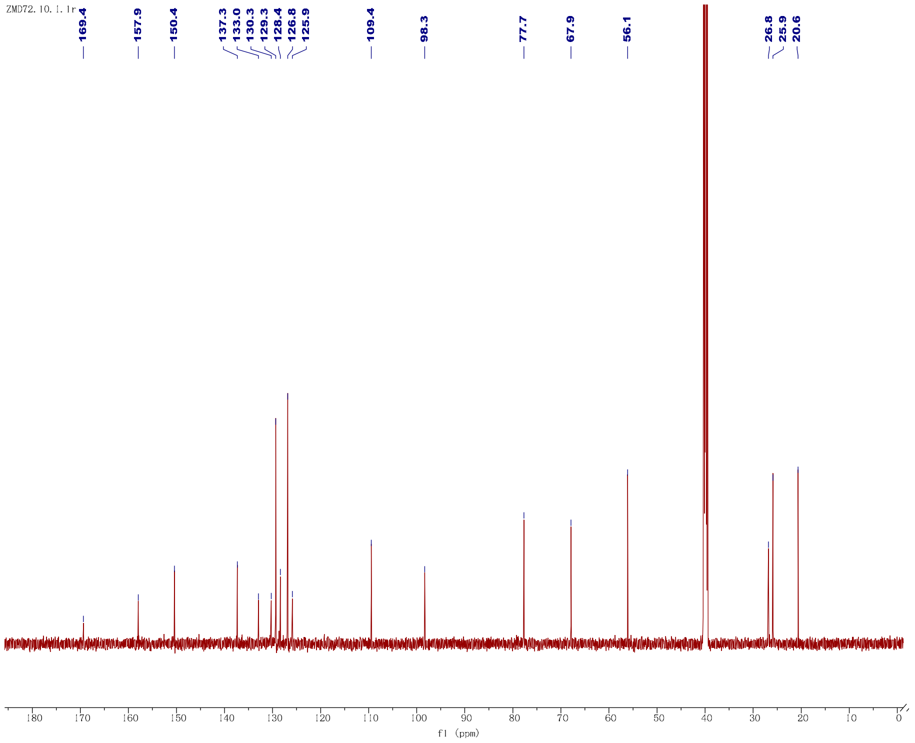

Figure S13: ^{13}C-NMR Spectrum of Compound **2**

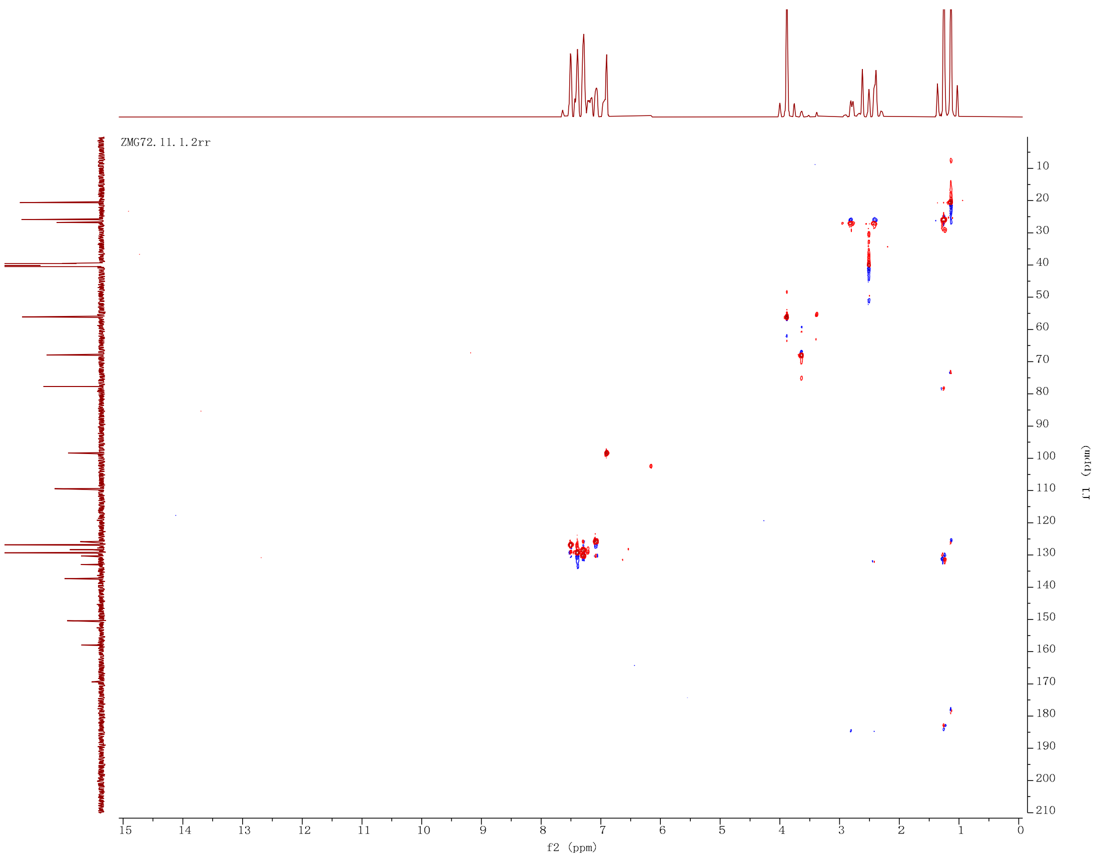

Figure S14: HSQC Spectrum of Compound **2**

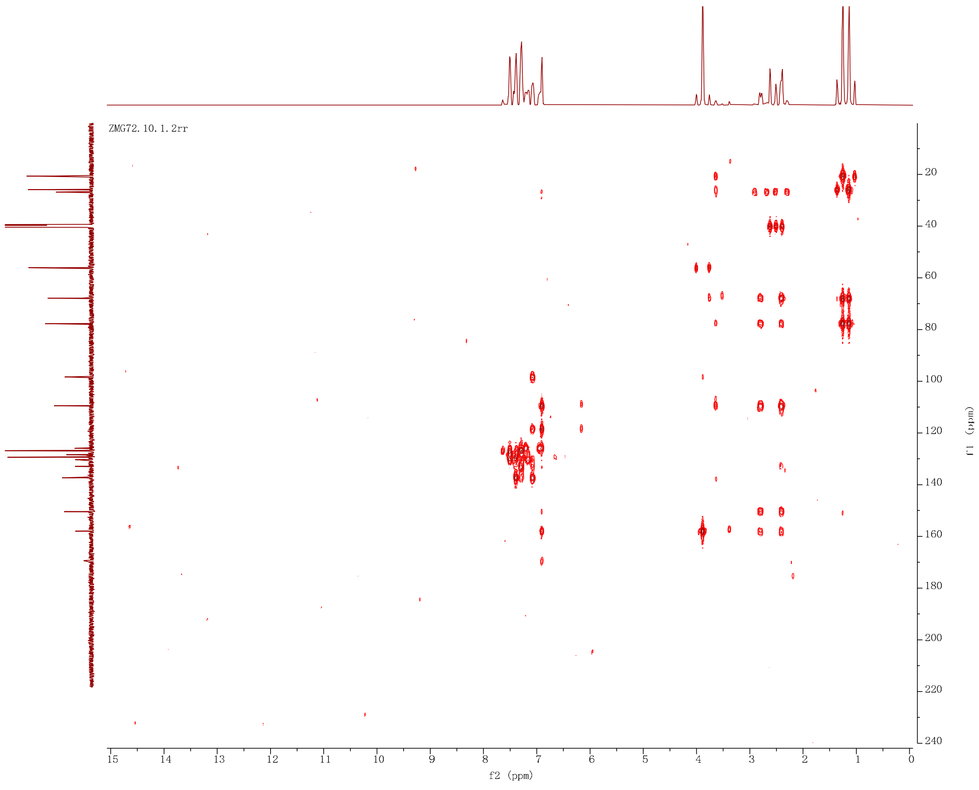

Figure S15: HMBC Spectrum of Compound **2**

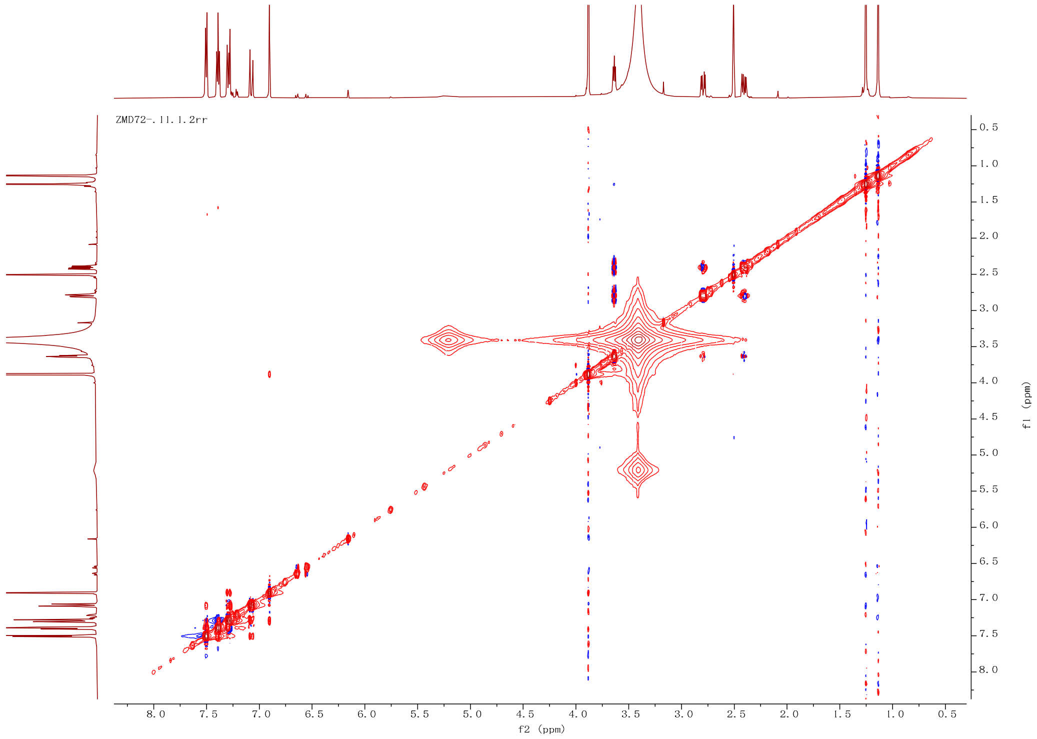

Figure S16: NOESY Spectrum of Compound **2**

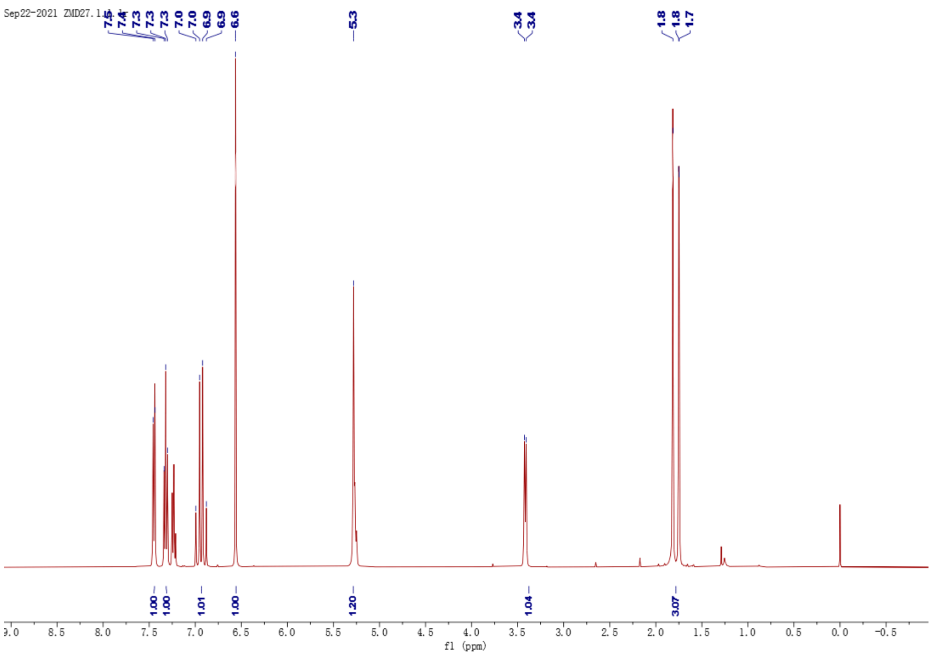

Figure S17: ^{1}H-NMR Spectrum of Compound **3**

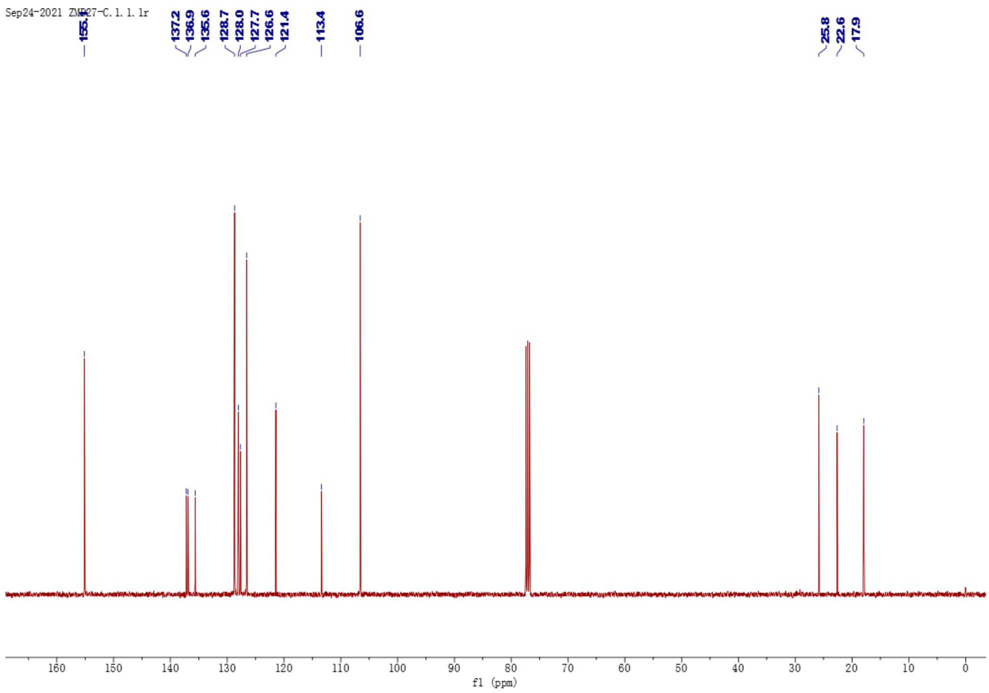

Figure S18: ^{13}C-NMR Spectrum of Compound **3**

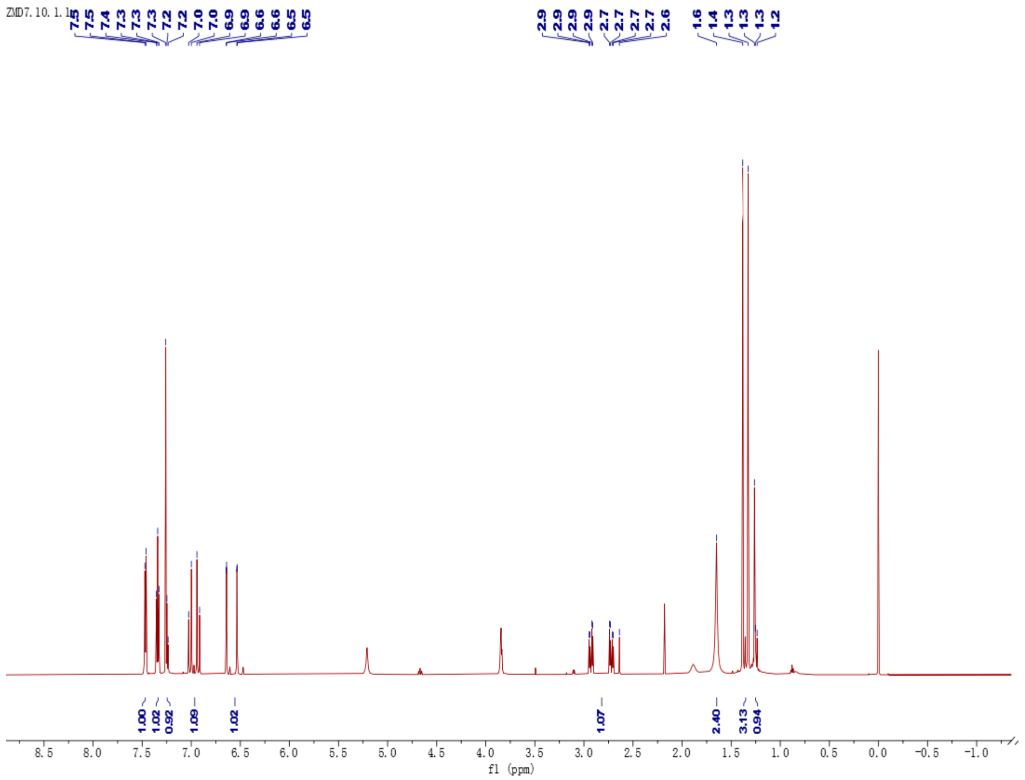

Figure S19: ^{1}H-NMR Spectrum of Compound **4**

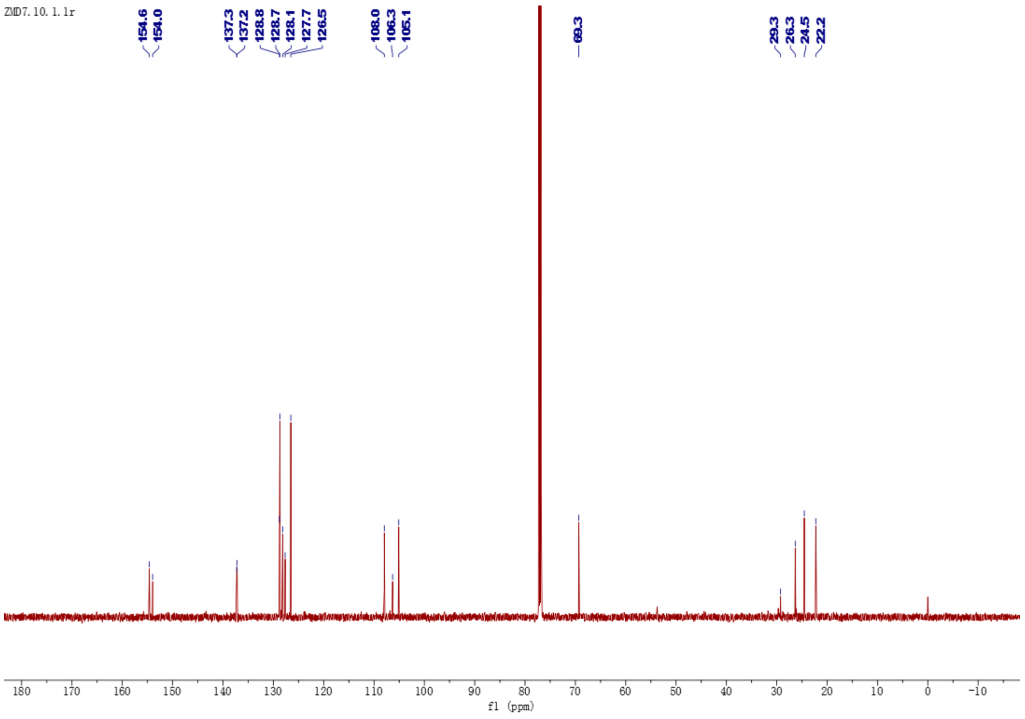

Figure S20: ^{13}C-NMR Spectrum of Compound **4**

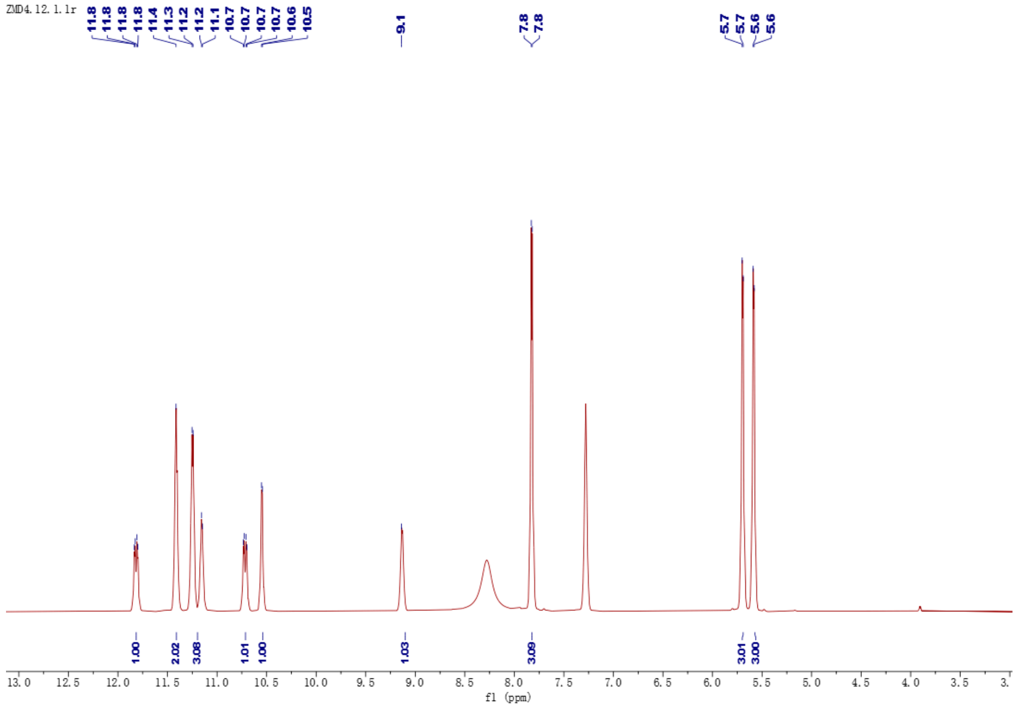

Figure S21: ^{1}H-NMR Spectrum of Compound **5**

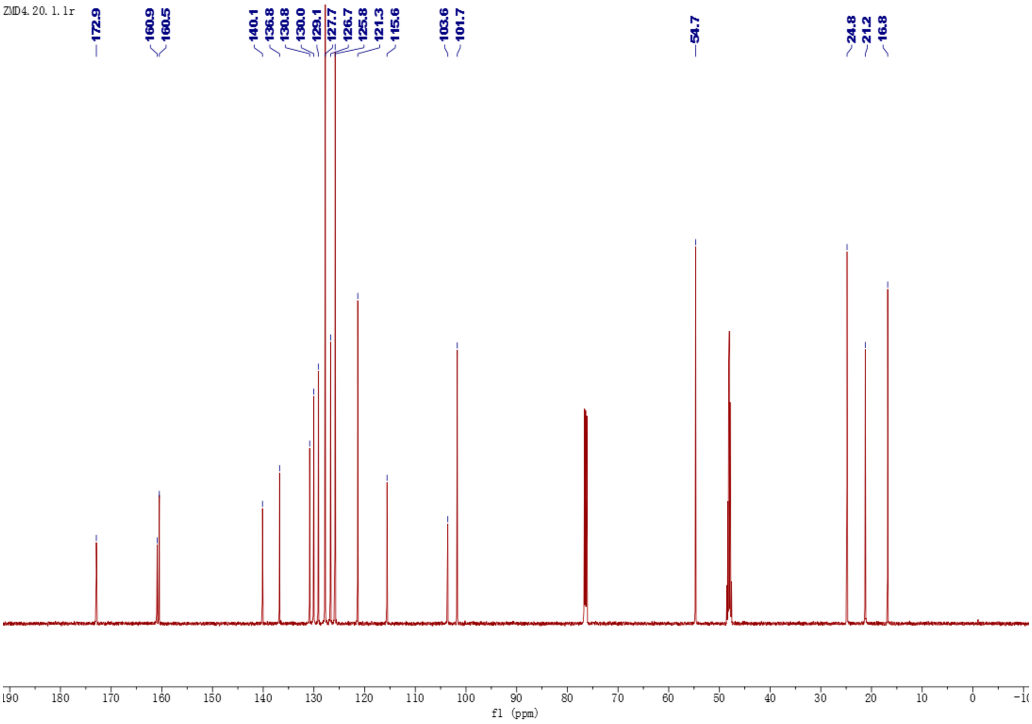

Figure S22: ^{13}C-NMR Spectrum of Compound **5**

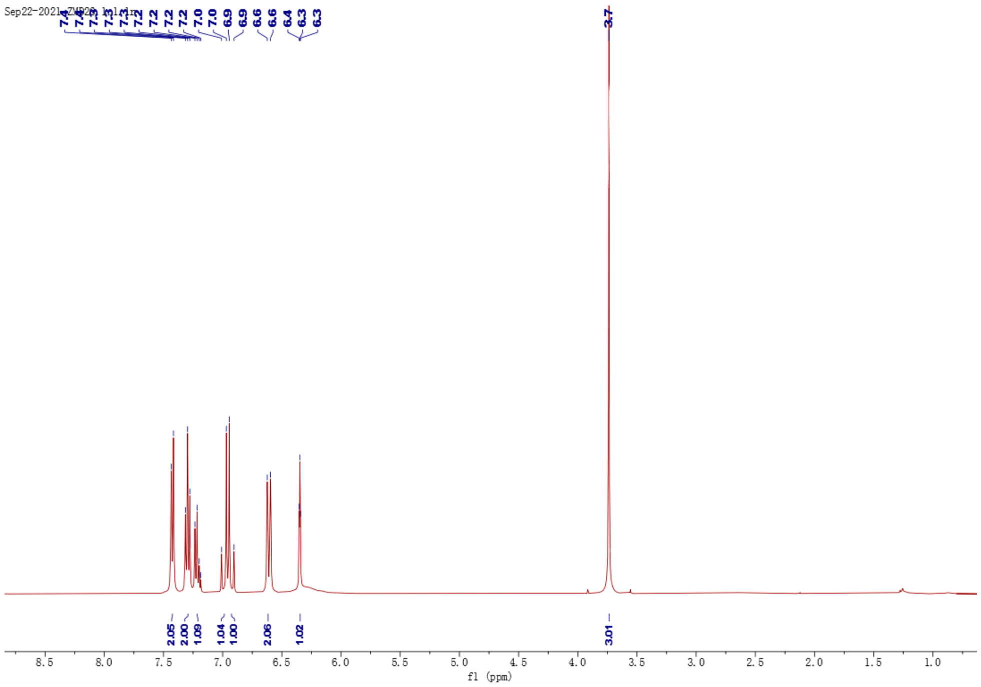

Figure S23: ^{1}H-NMR Spectrum of Compound **6**

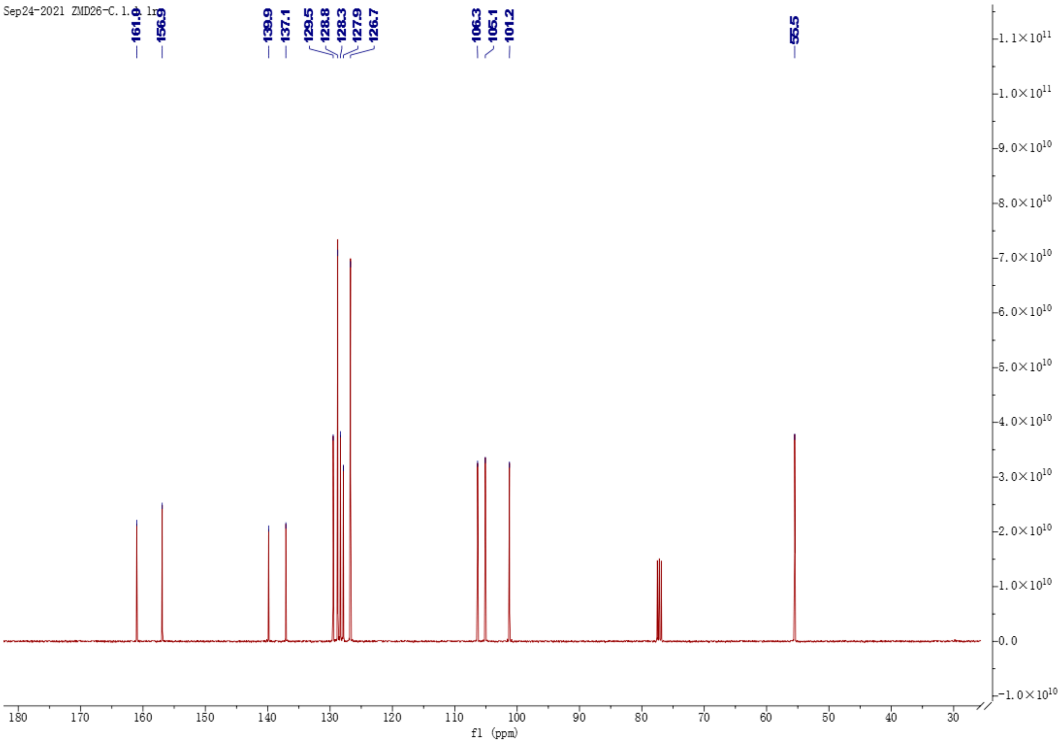

Figure S24: ^{13}C-NMR Spectrum of Compound **6**

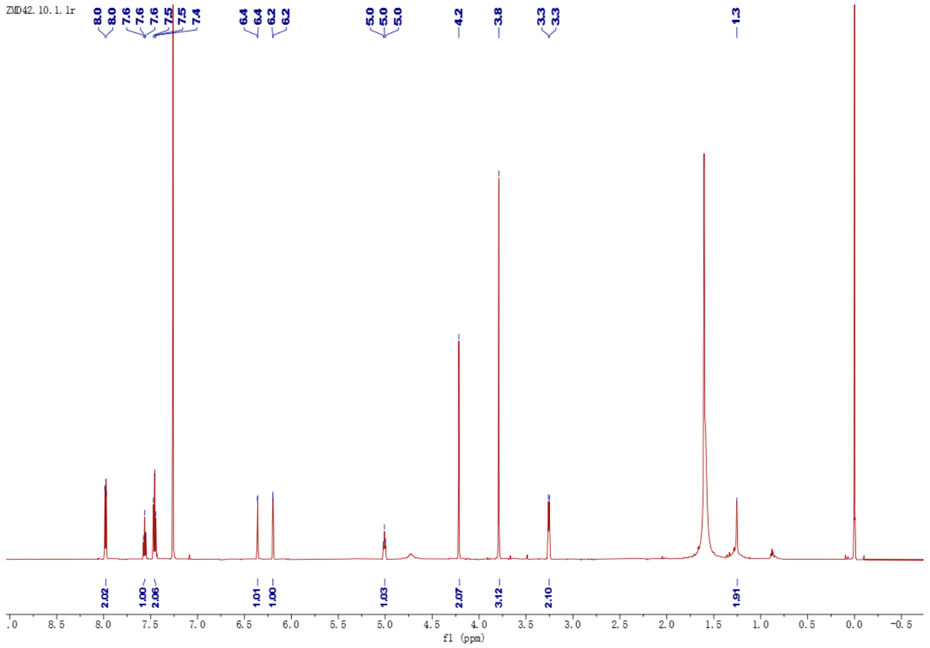

Figure S25: ^{1}H-NMR Spectrum of Compound **7**

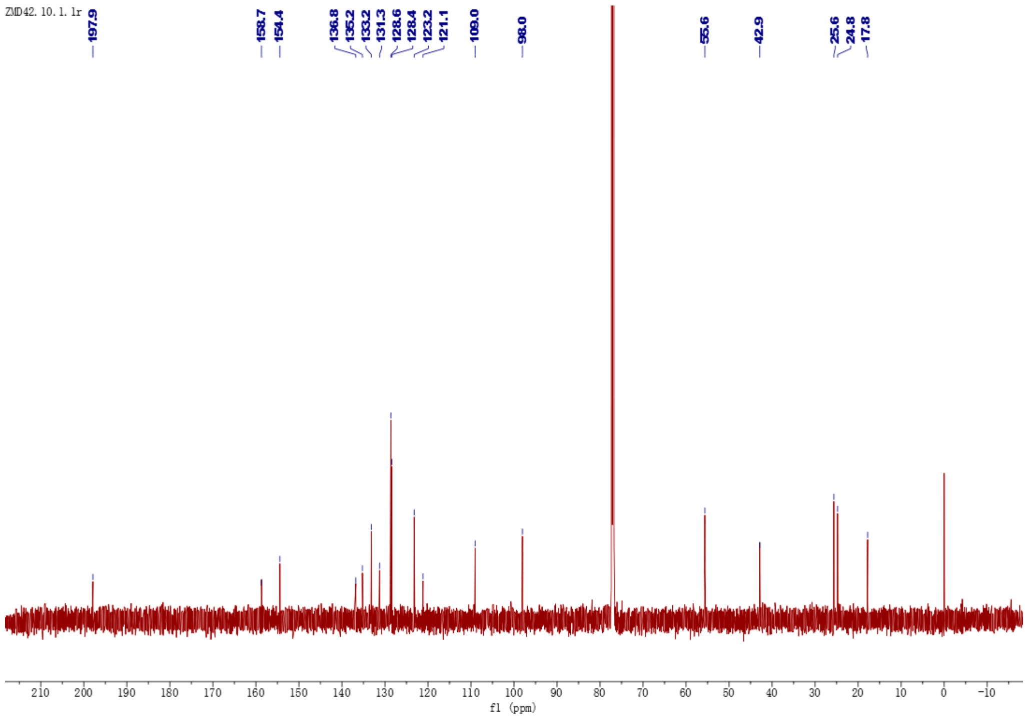

Figure S26: ^{13}C-NMR Spectrum of Compound **7**

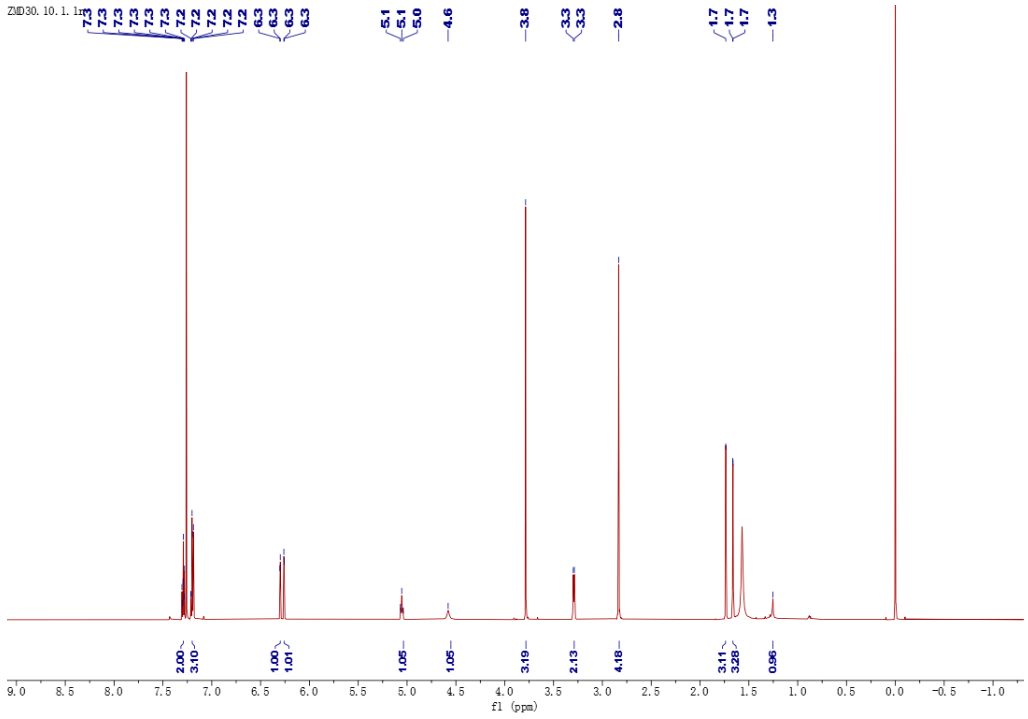

Figure S27: ^{1}H-NMR Spectrum of Compound **8**

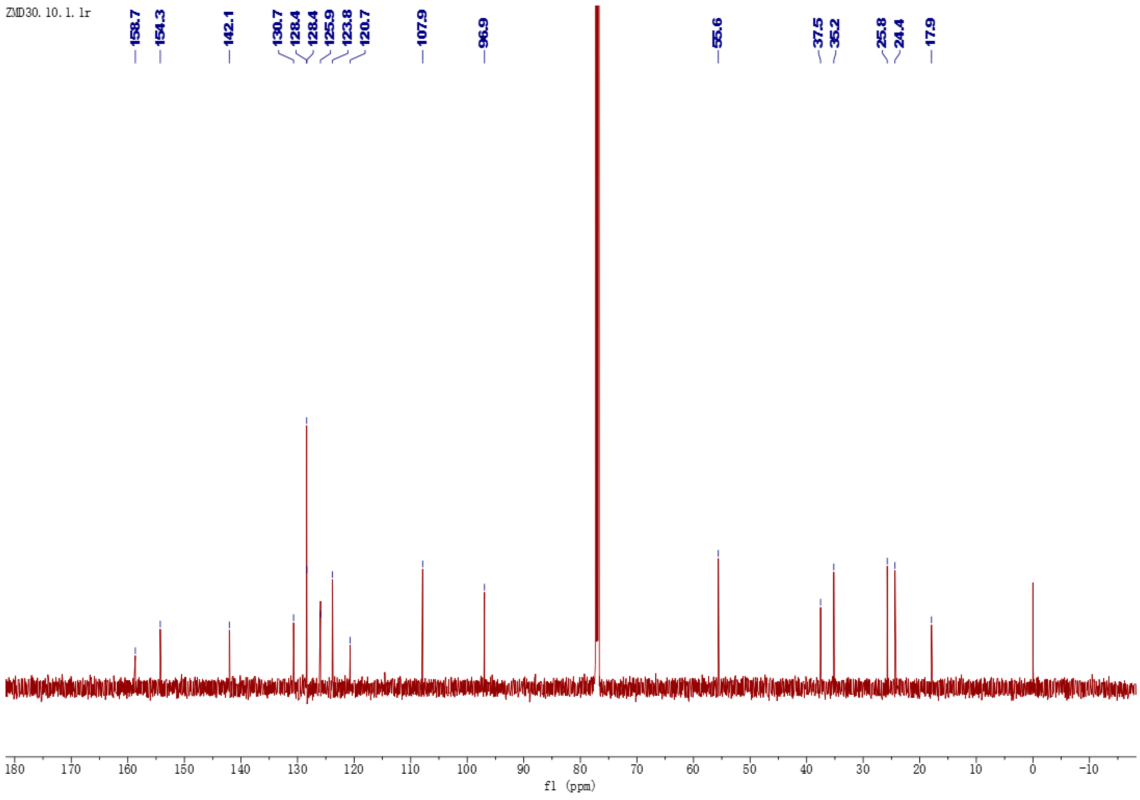

Figure S28: ^{13}C-NMR Spectrum of Compound **8**

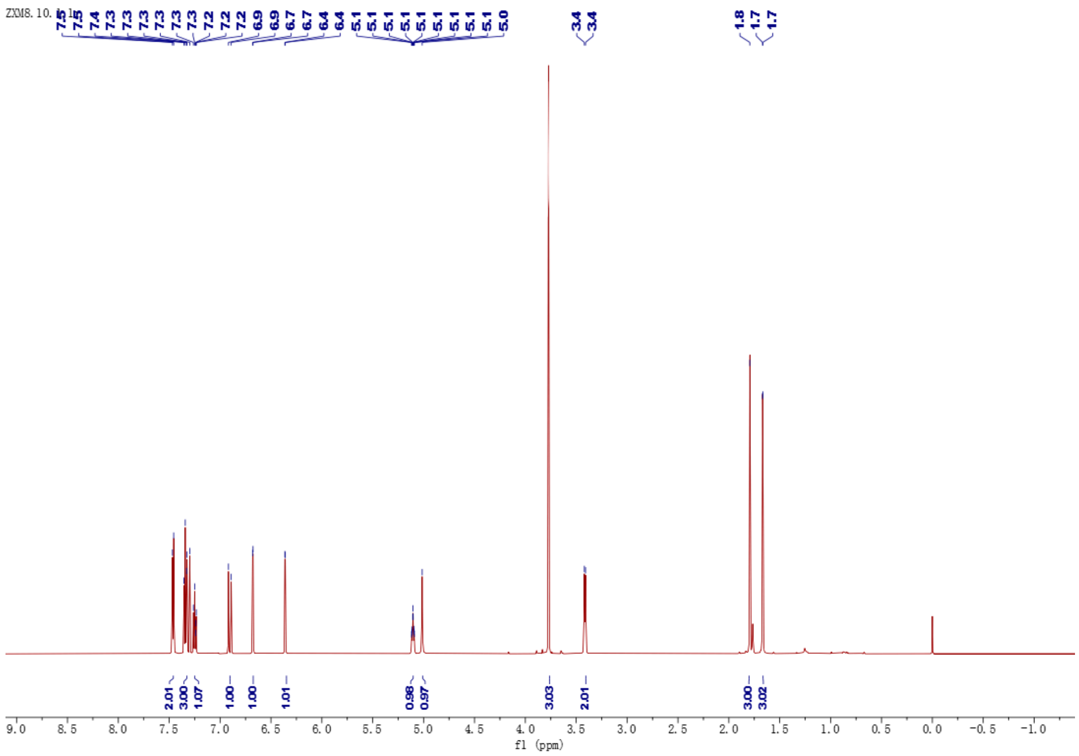

Figure S29: ^{1}H-NMR Spectrum of Compound **9**

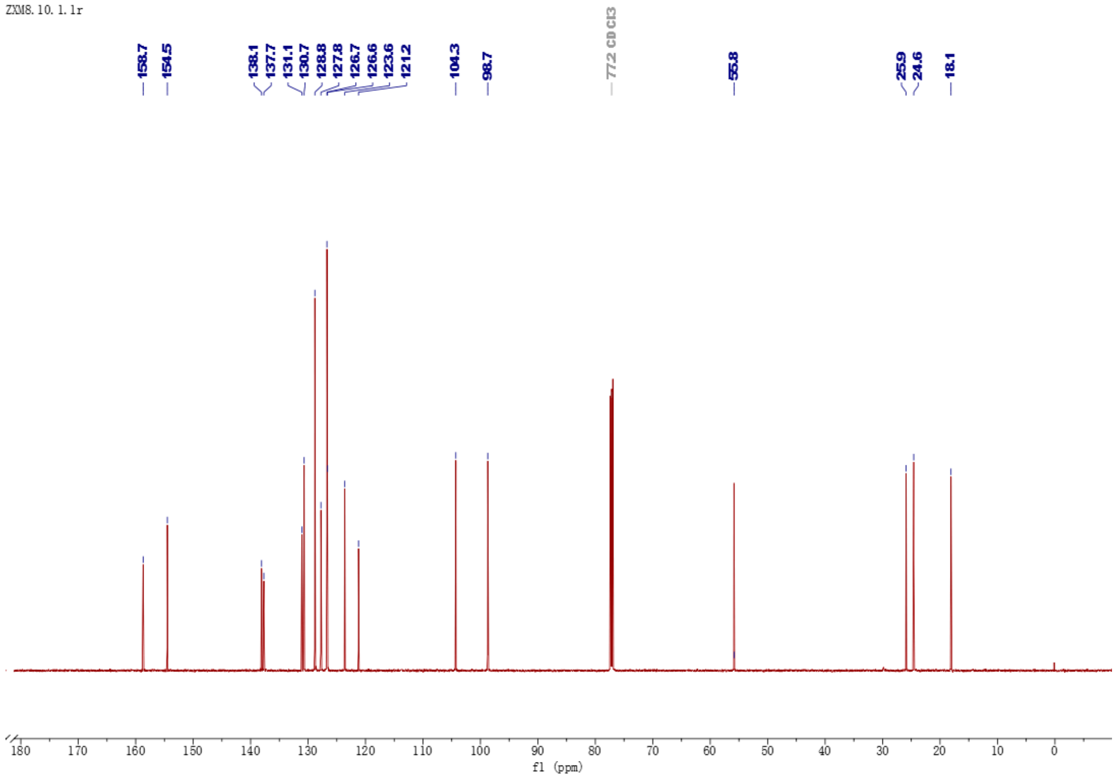

Figure S30: ^{13}C-NMR Spectrum of Compound **9**

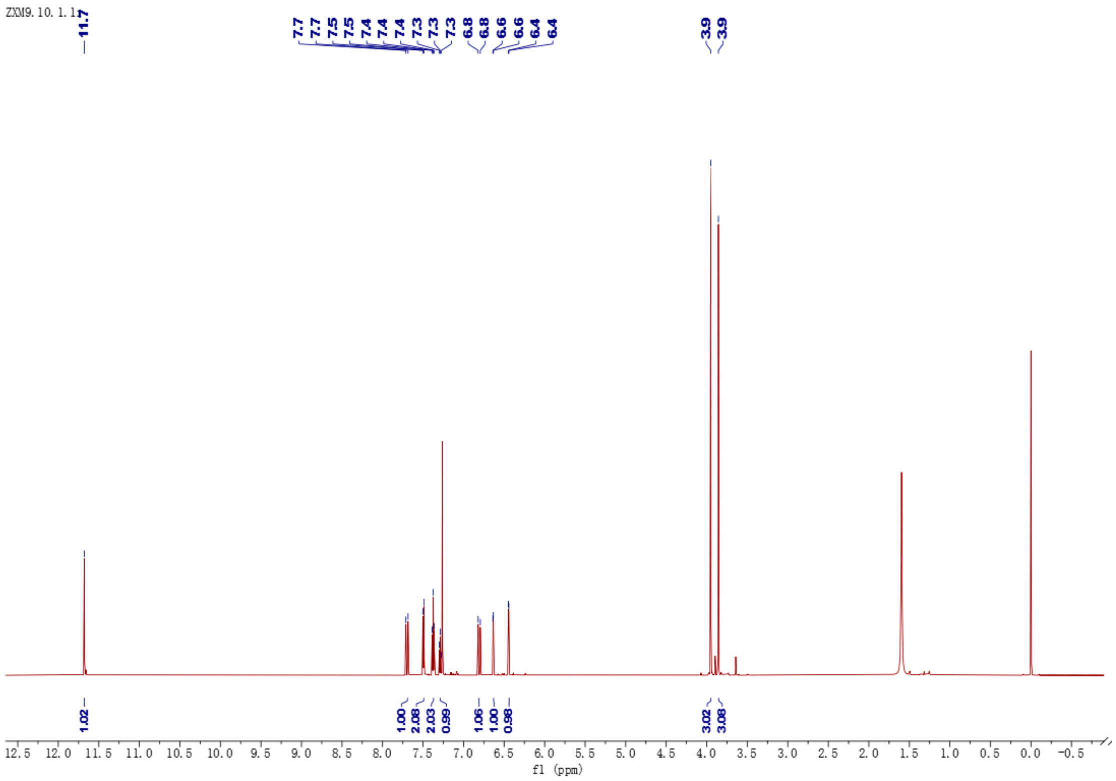

Figure S31: ^{1}H-NMR Spectrum of Compound **10**

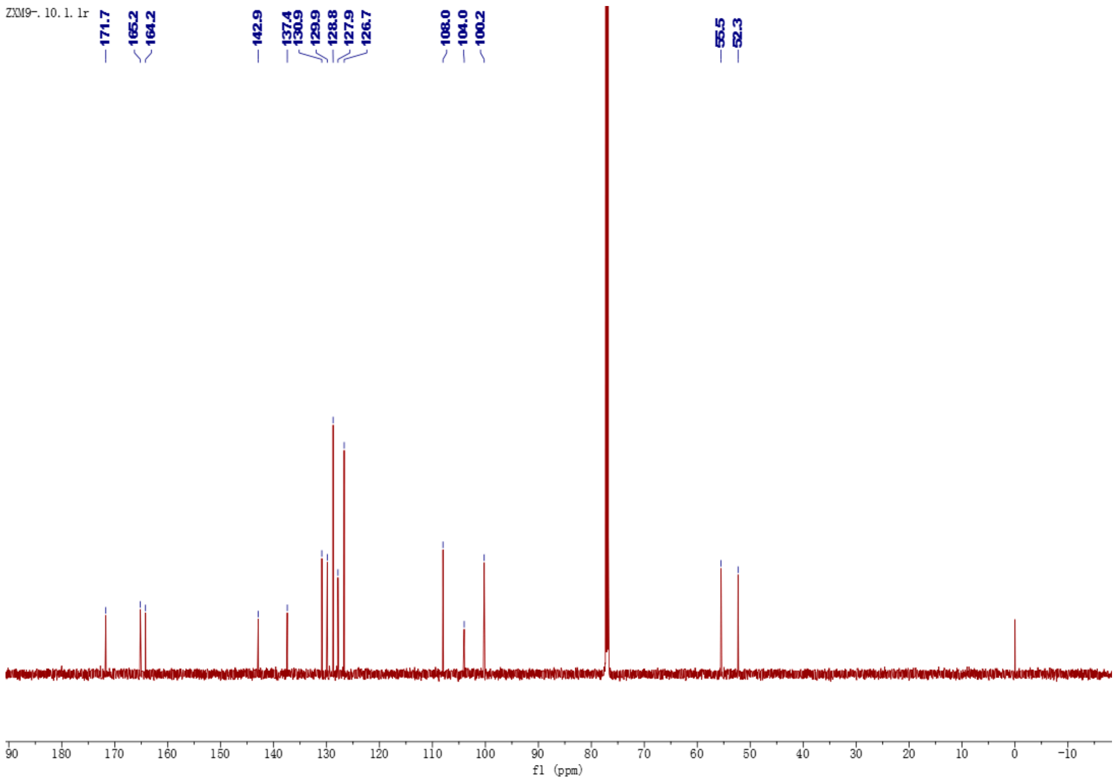

Figure S32: ^{13}C-NMR Spectrum of Compound **10**

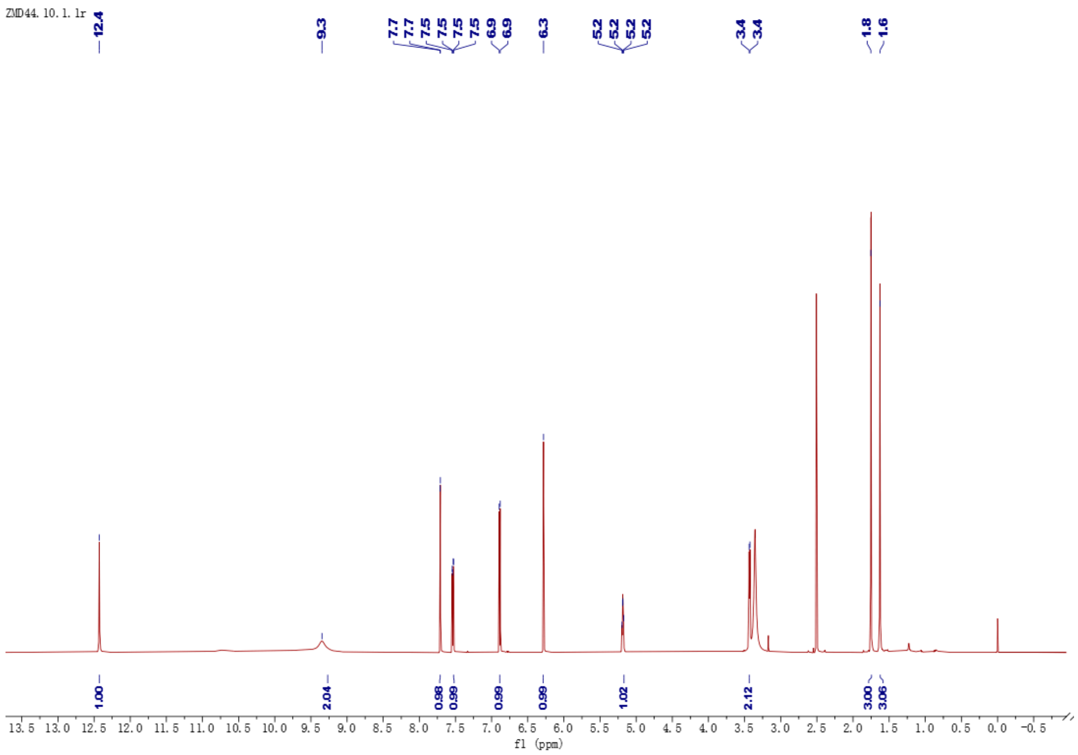

Figure S33: ^{1}H-NMR Spectrum of Compound **11**

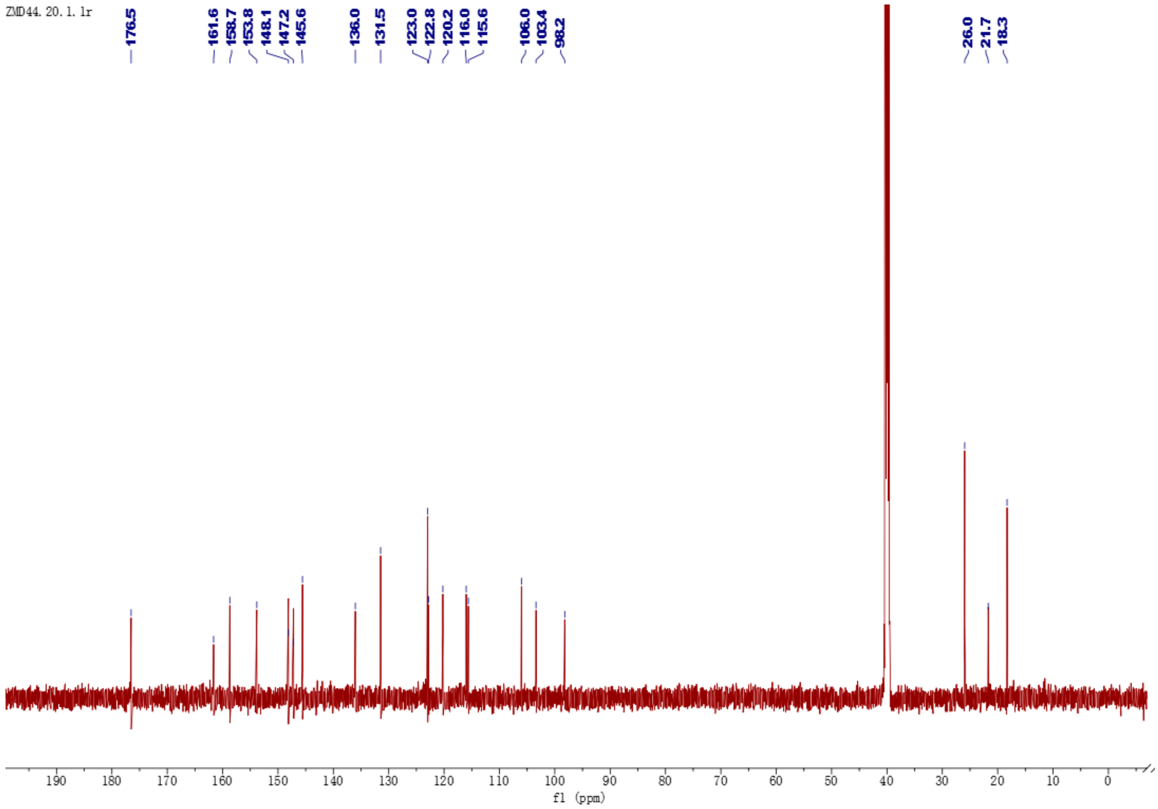

Figure S34: ^{13}C-NMR Spectrum of Compound **11**

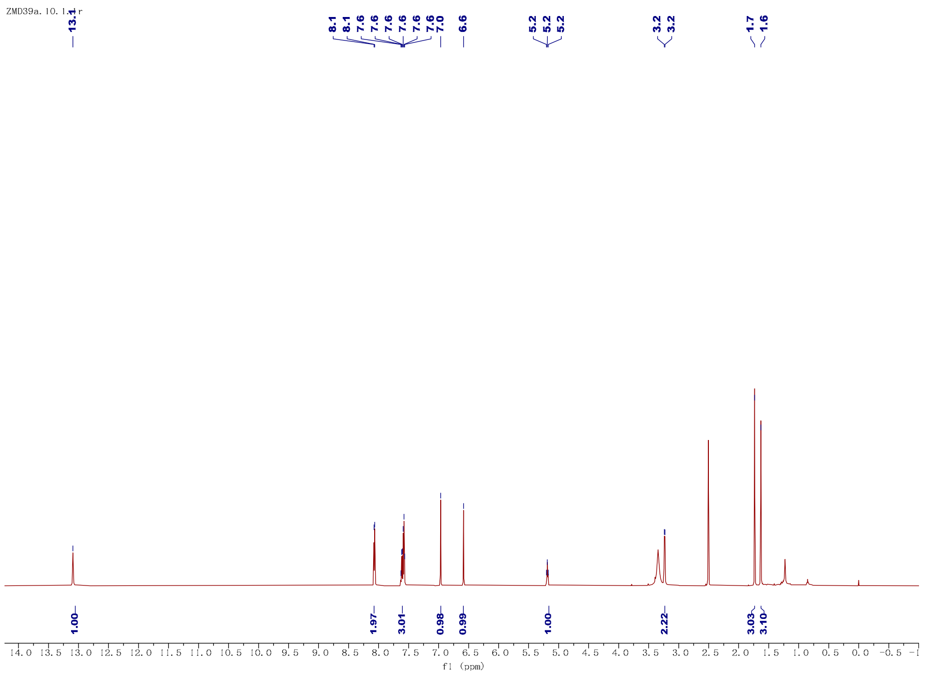

Figure S35: ^{1}H-NMR Spectrum of Compound **12**

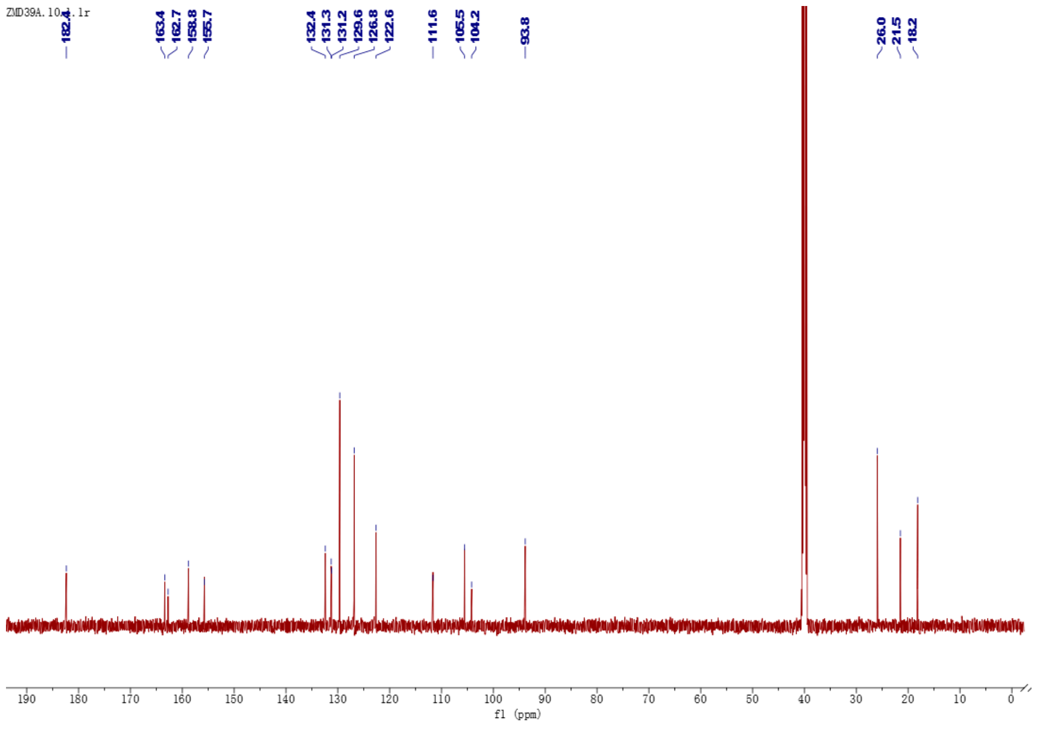

Figure S36: ^{13}C-NMR Spectrum of Compound **12**

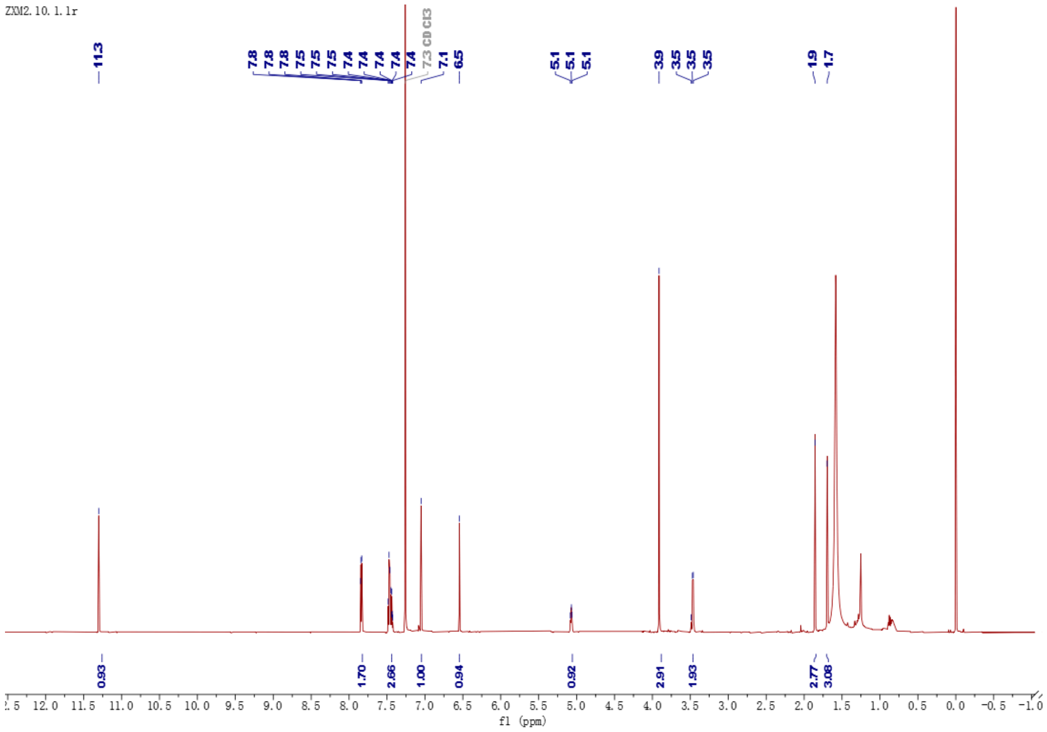

Figure S37: ^{1}H-NMR Spectrum of Compound **13**

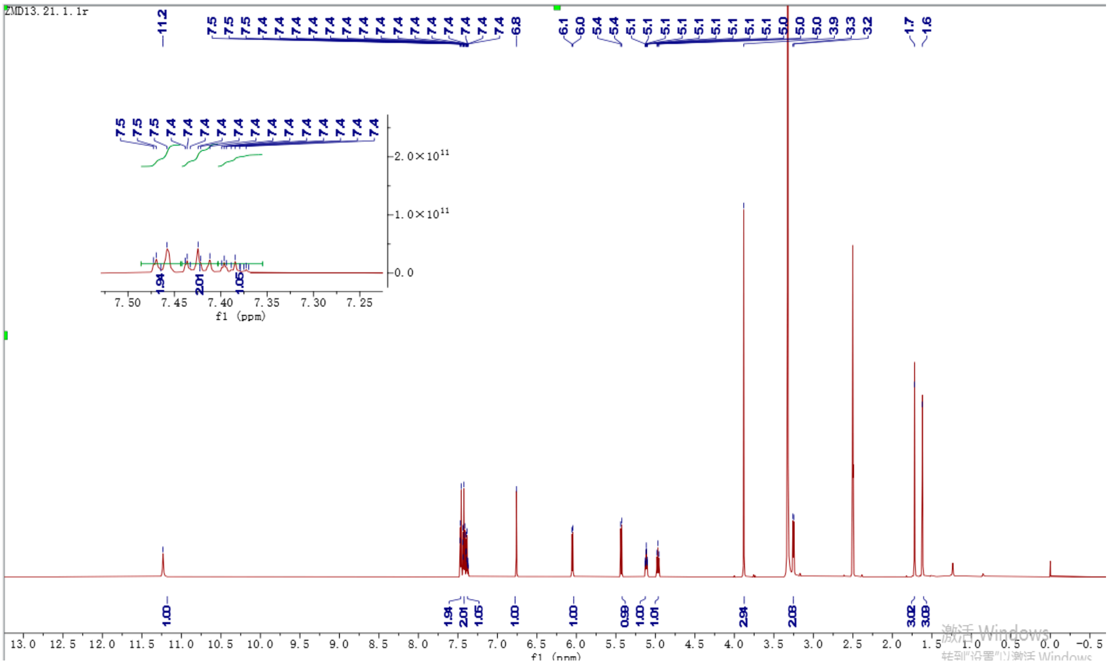

Figure S38: ^{1}H-NMR Spectrum of Compound **14**

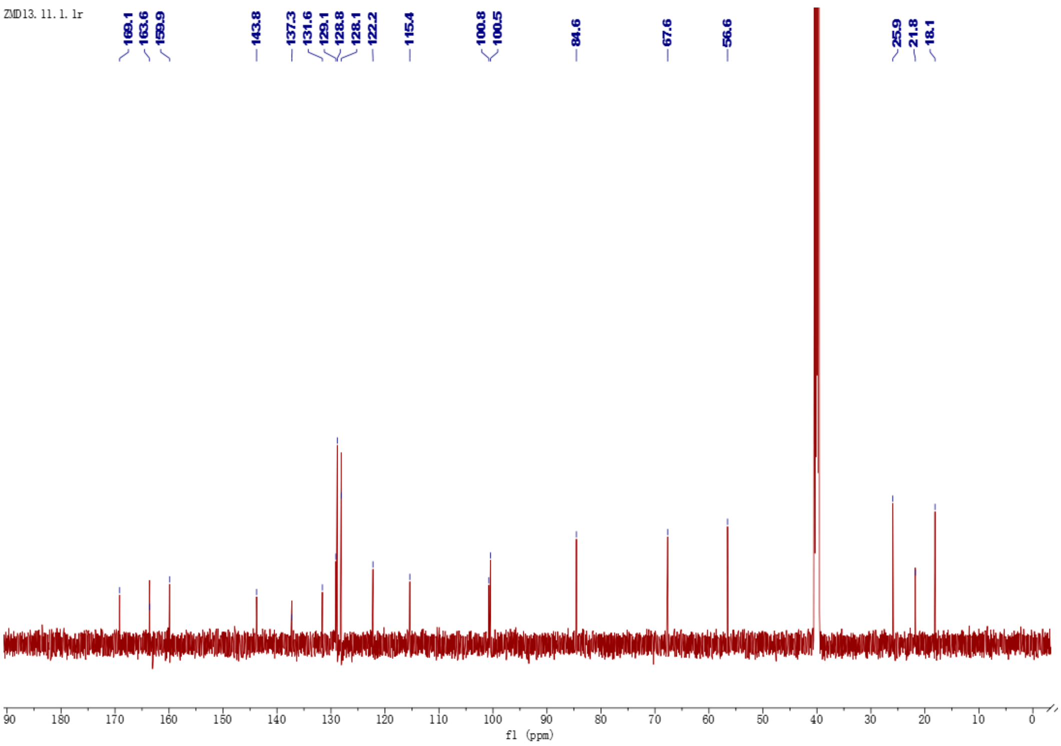

Figure S39: ^{13}C-NMR Spectrum of Compound **14**

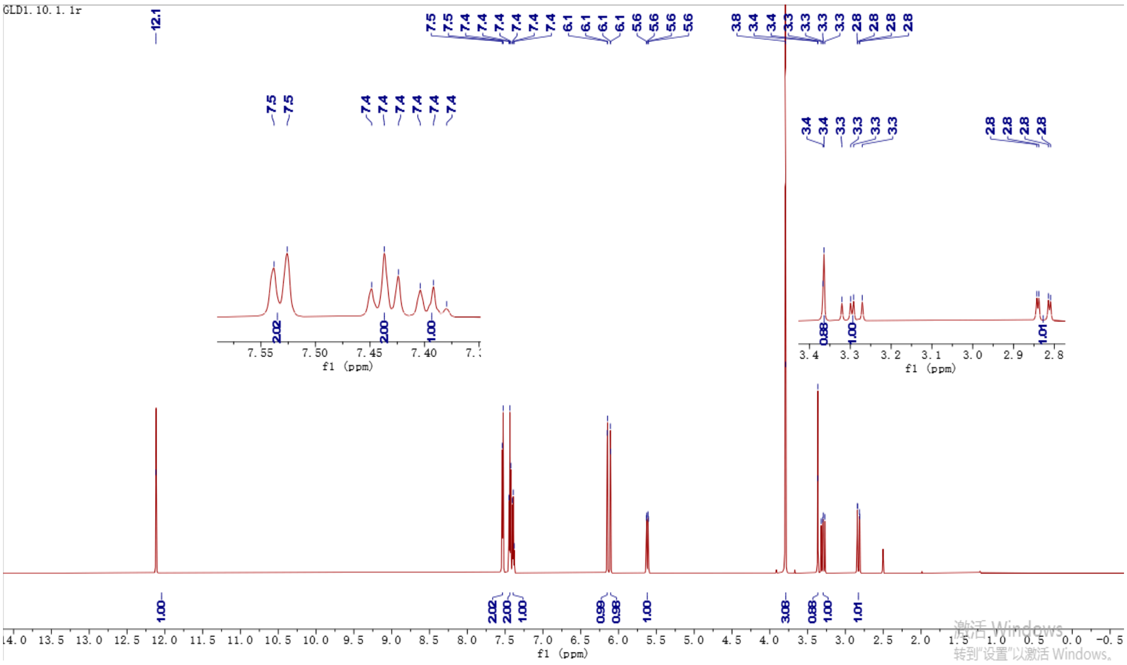

Figure S40: ^{1}H-NMR Spectrum of Compound **15**

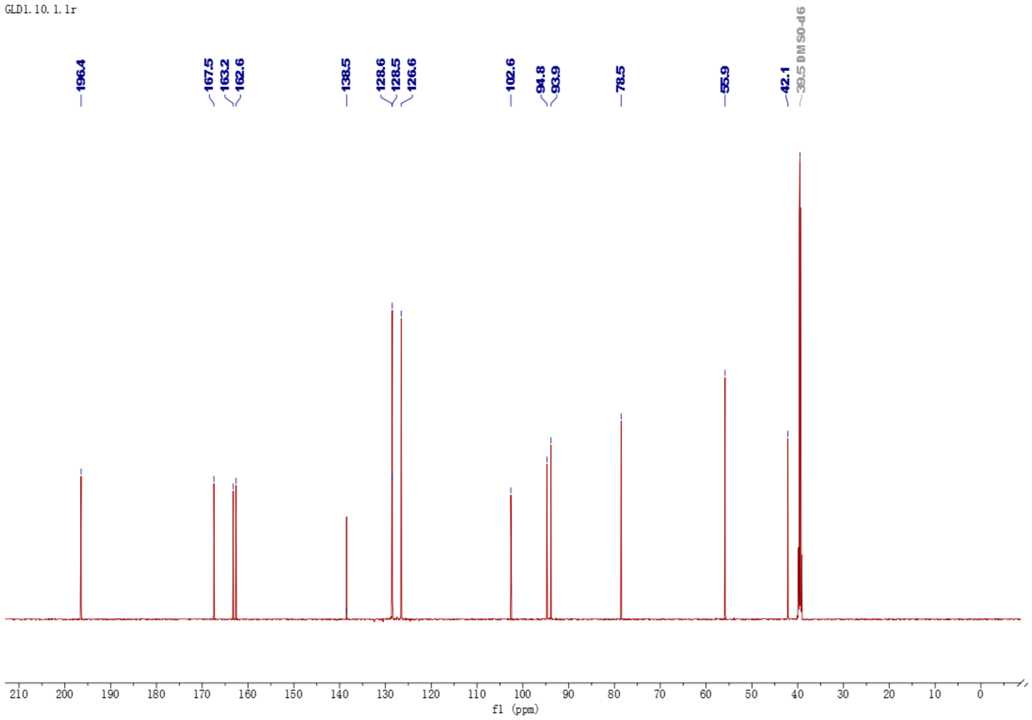

Figure S41: ^{13}C-NMR Spectrum of Compound **15**

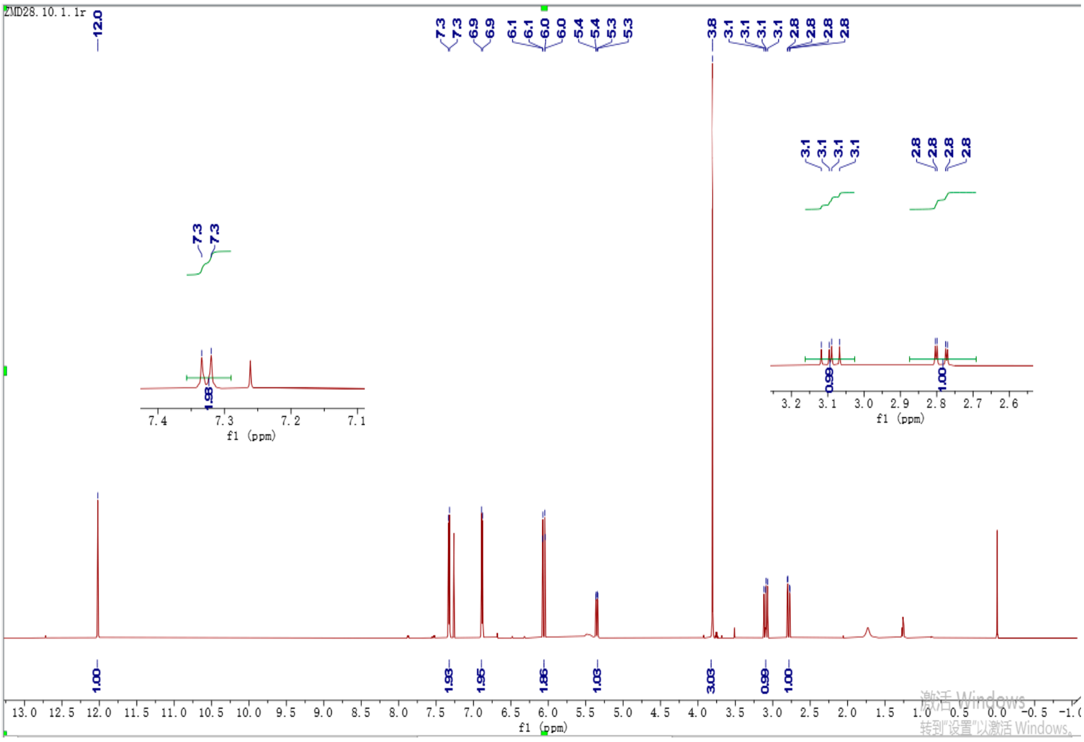

Figure S42: ^{1}H-NMR Spectrum of Compound **16**

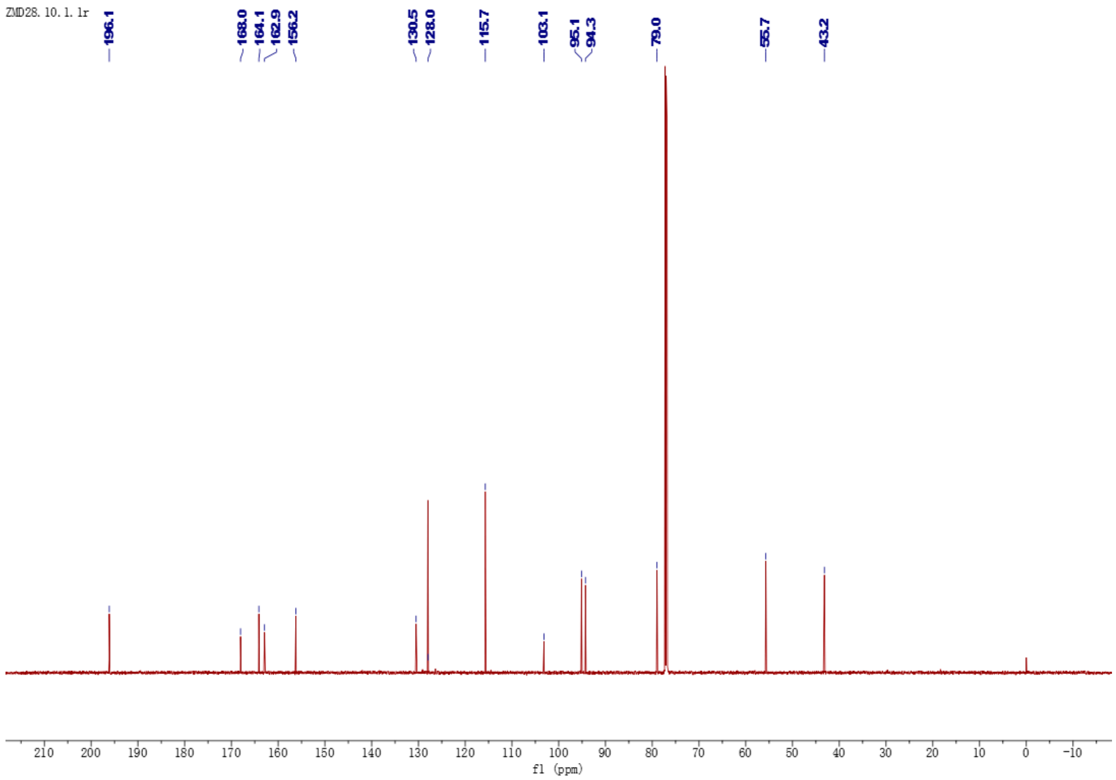

Figure S43: ^{13}C-NMR Spectrum of Compound **16**

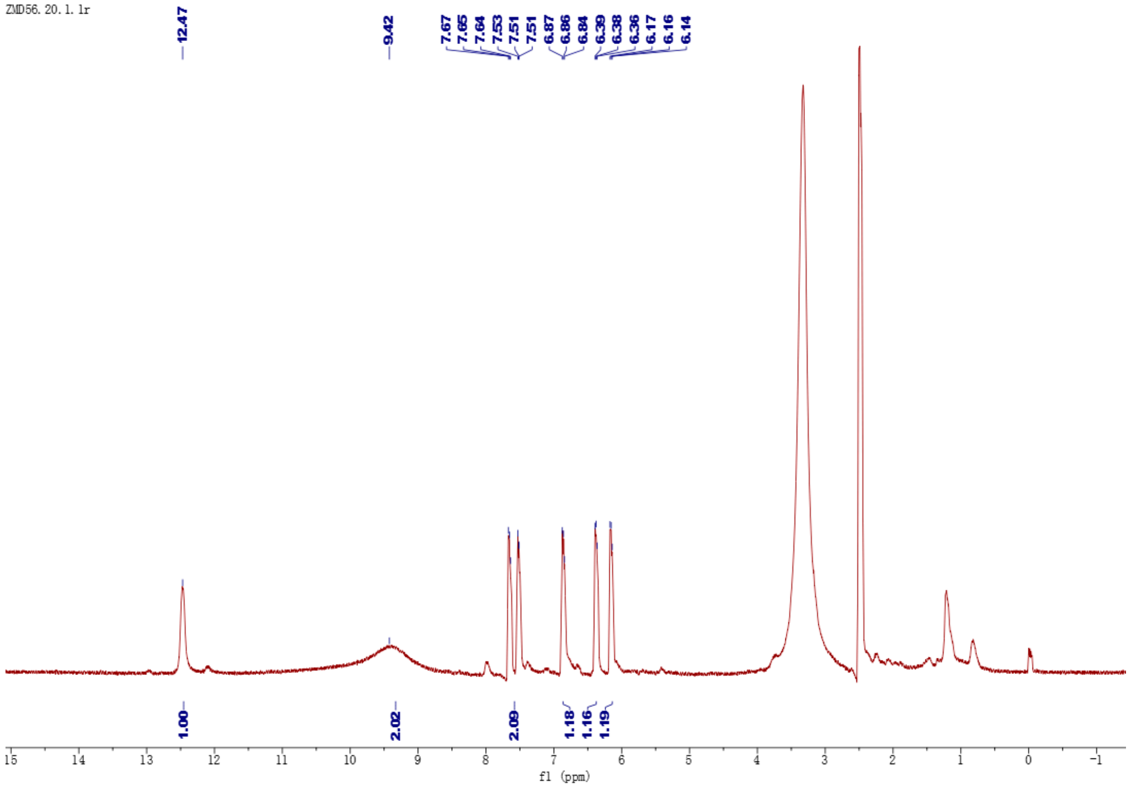

Figure S44: ^{1}H-NMR Spectrum of Compound **17**

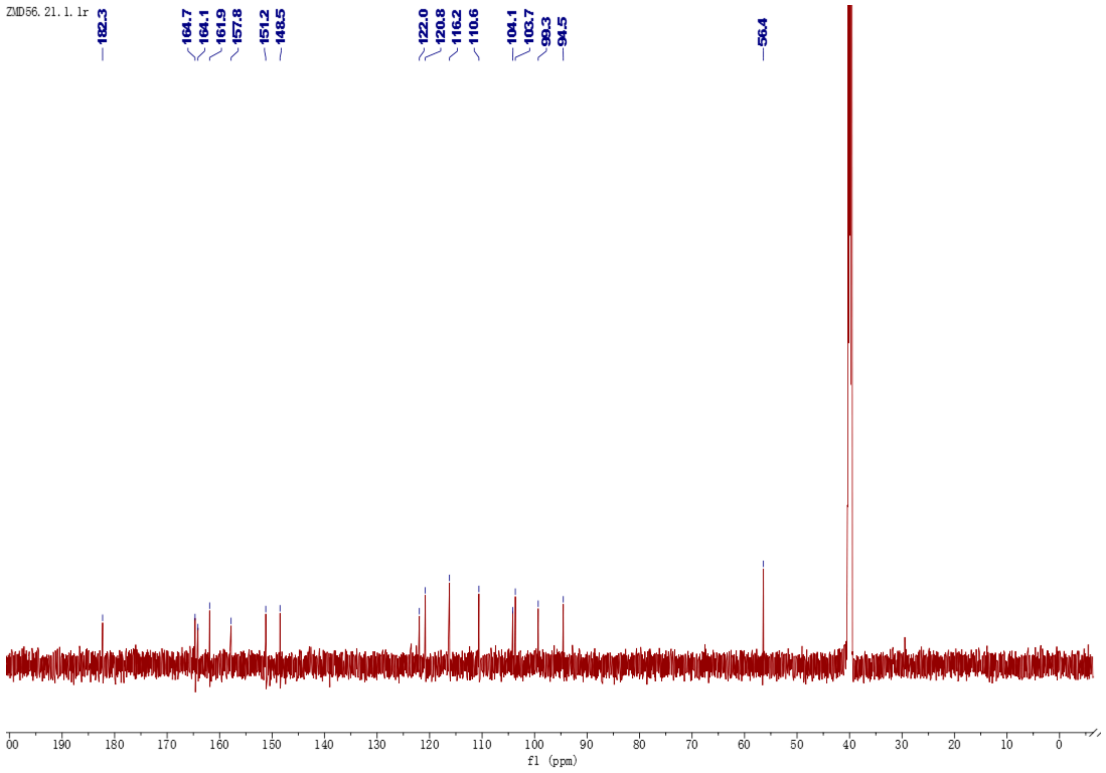

Figure S45: ^{13}C-NMR Spectrum of Compound **17**

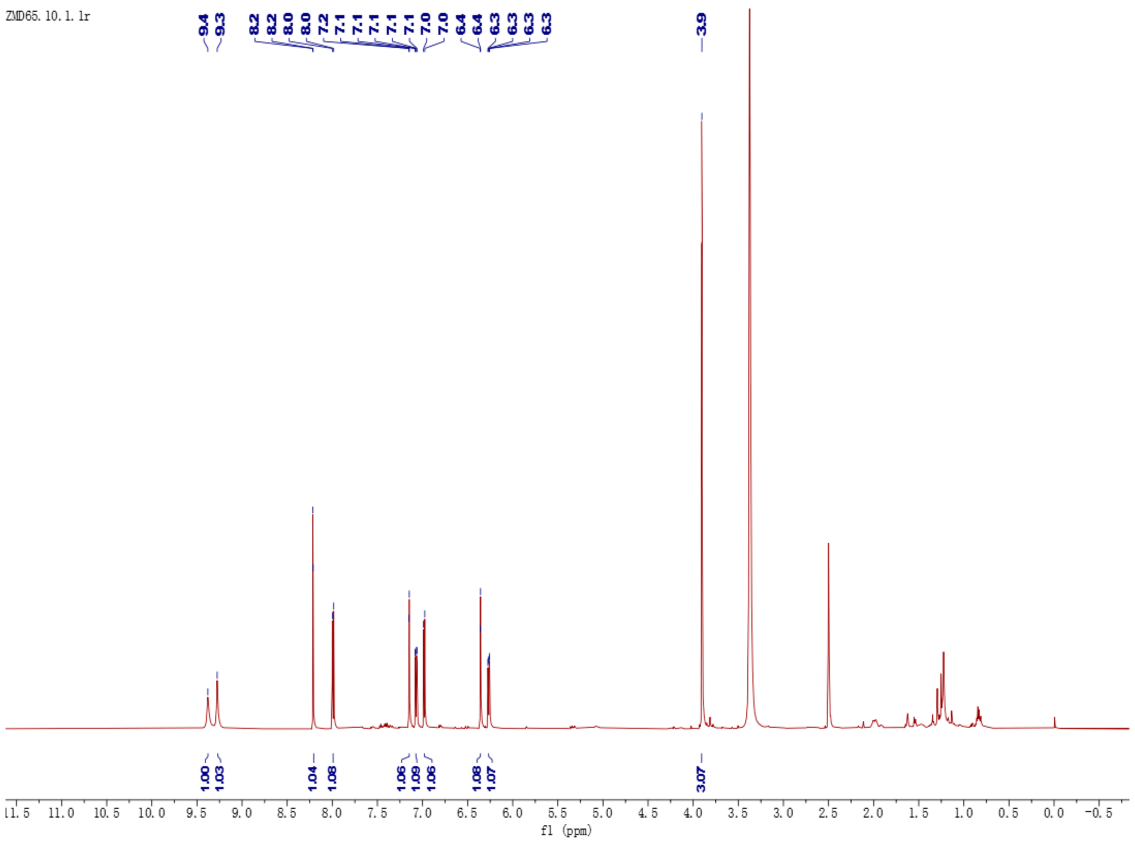

Figure S46: ^{1}H-NMR Spectrum of Compound **18**

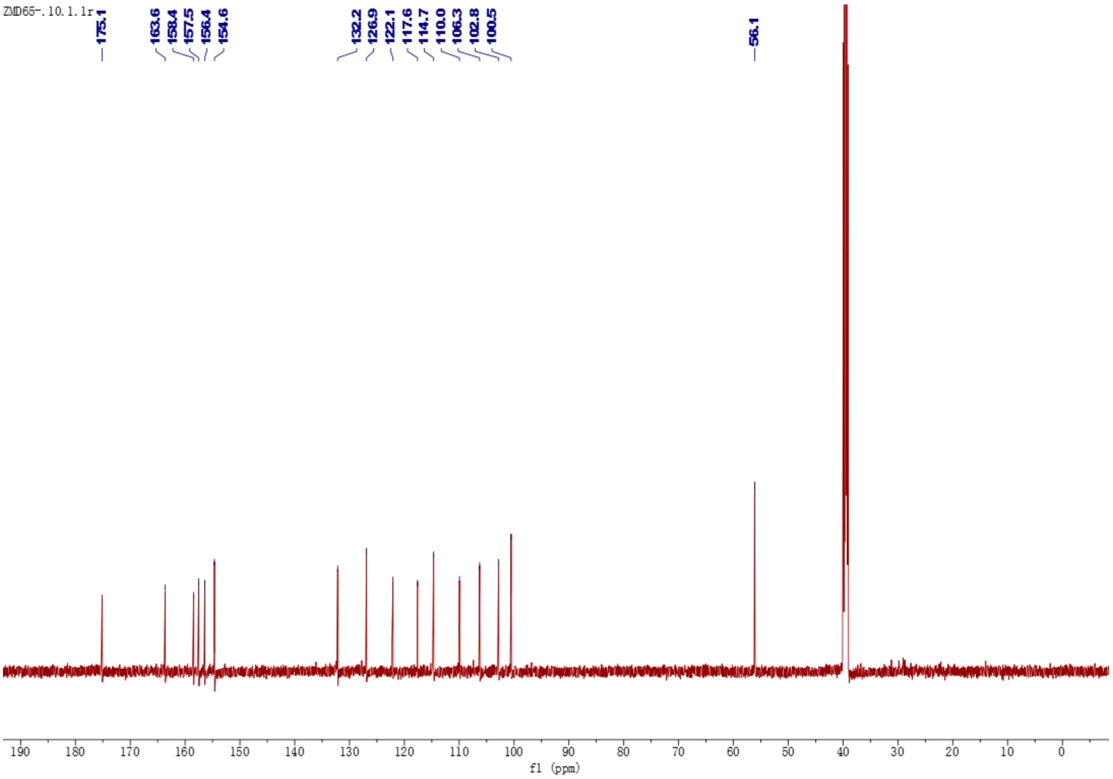

Figure S47: ^{13}C-NMR Spectrum of Compound **18**

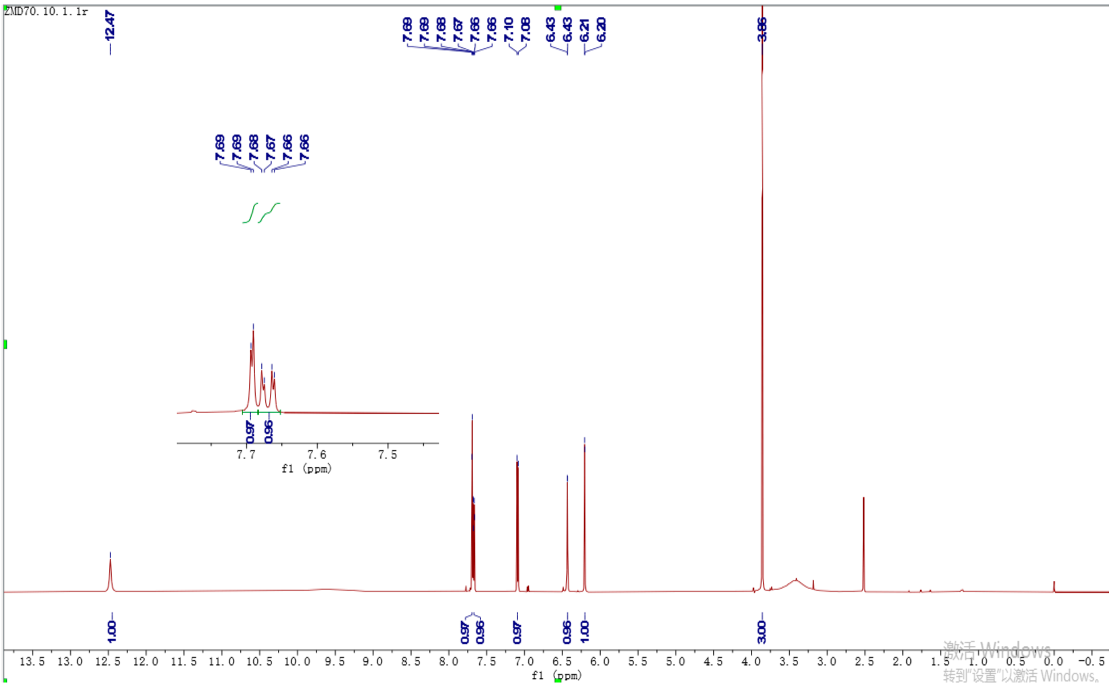

Figure S48: ^{1}H-NMR Spectrum of Compound **19**

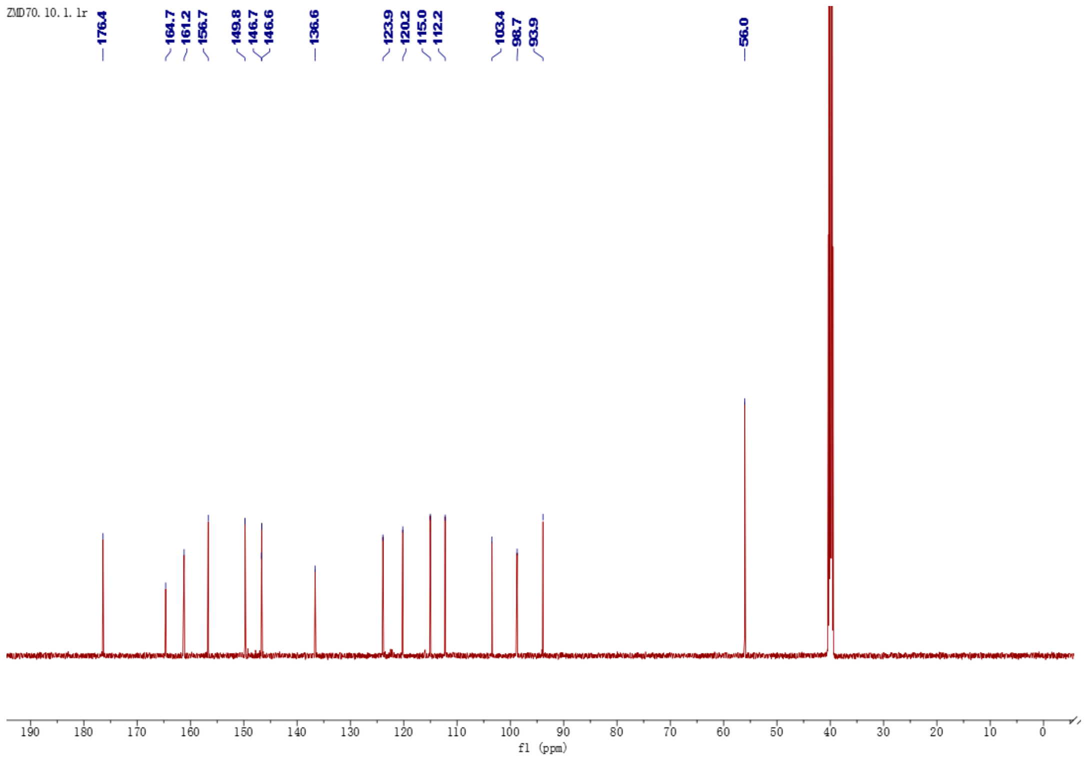

Figure S49: ^{13}C-NMR Spectrum of Compound **19**

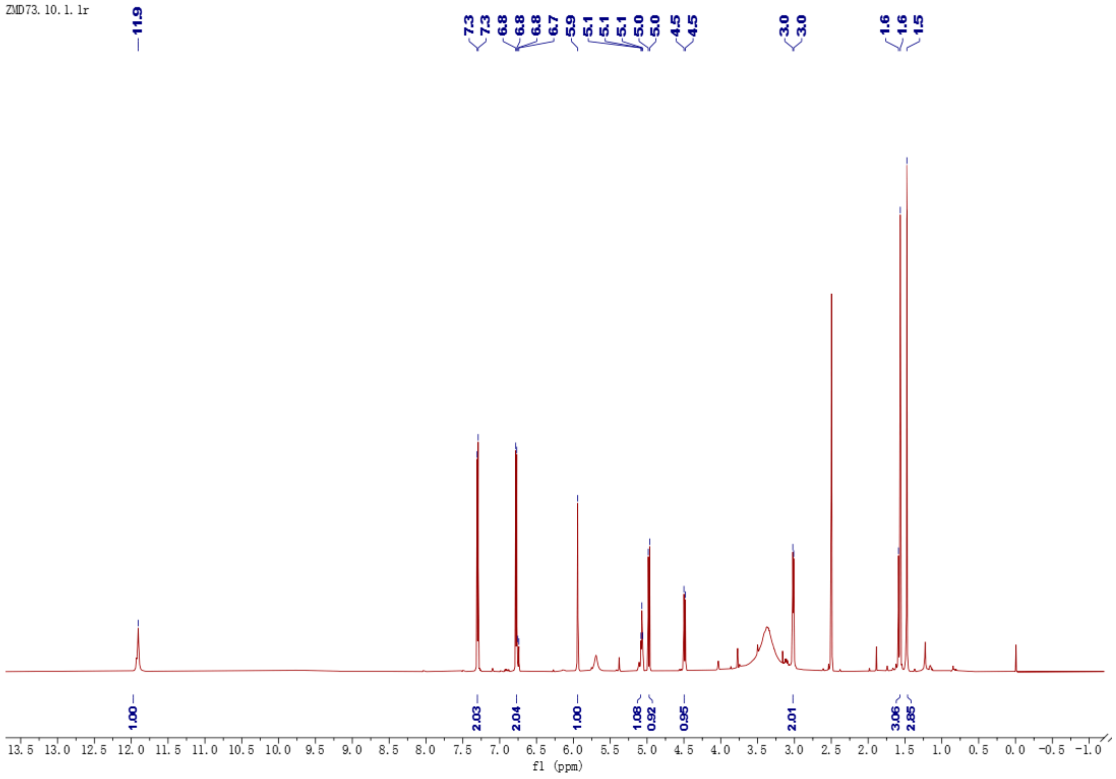

Figure S50: ^{1}H-NMR Spectrum of Compound **20**

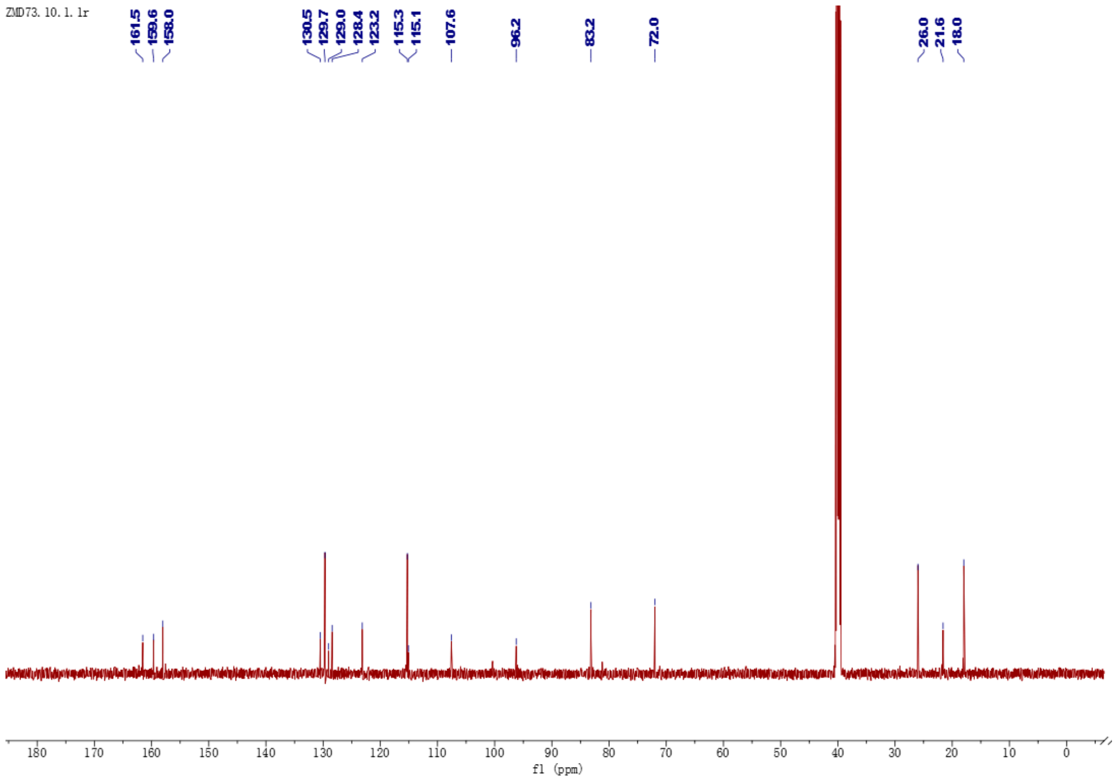

Figure S51: ^{13}C-NMR Spectrum of Compound **20**

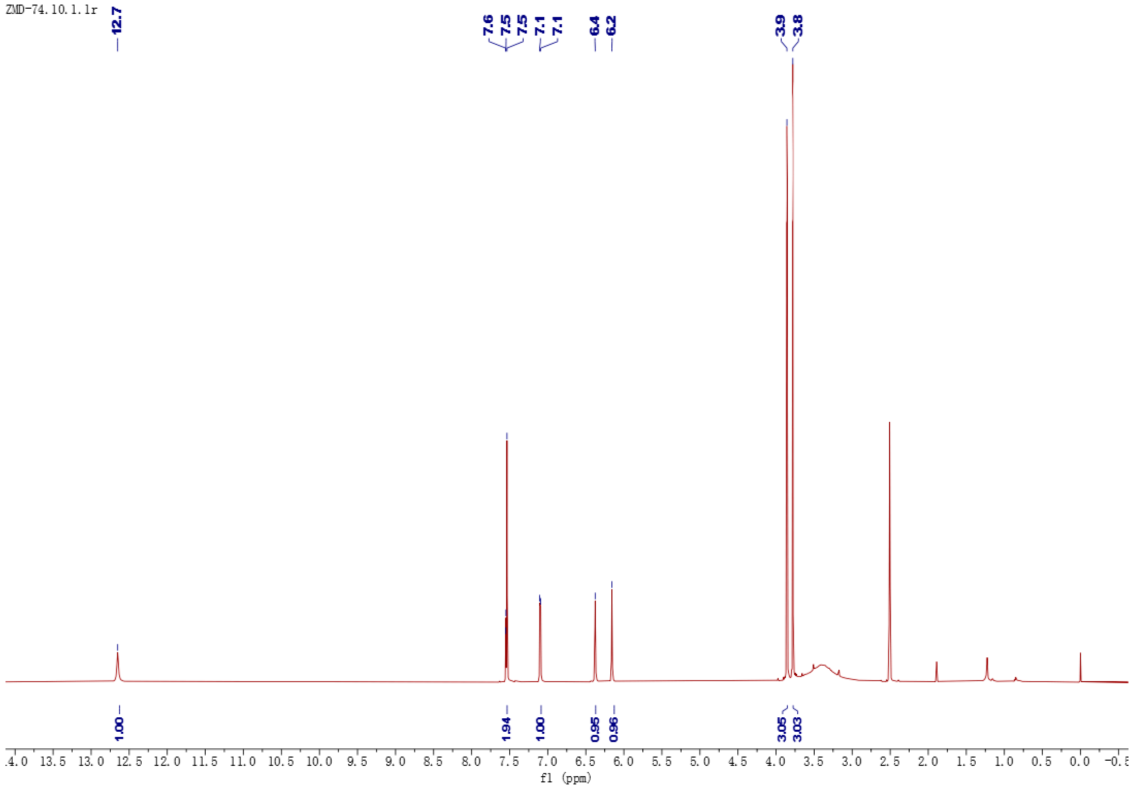

Figure S52: ^{1}H-NMR Spectrum of Compound **21**

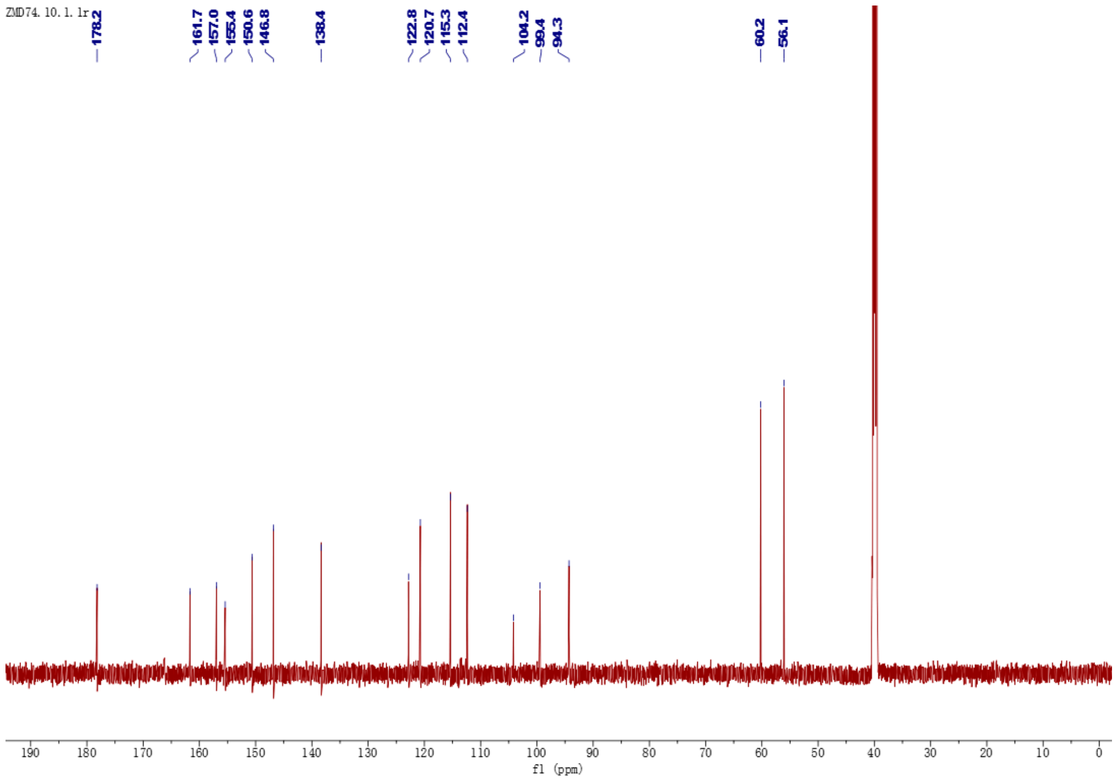

Figure S53: ^{13}C-NMR Spectrum of Compound **21**

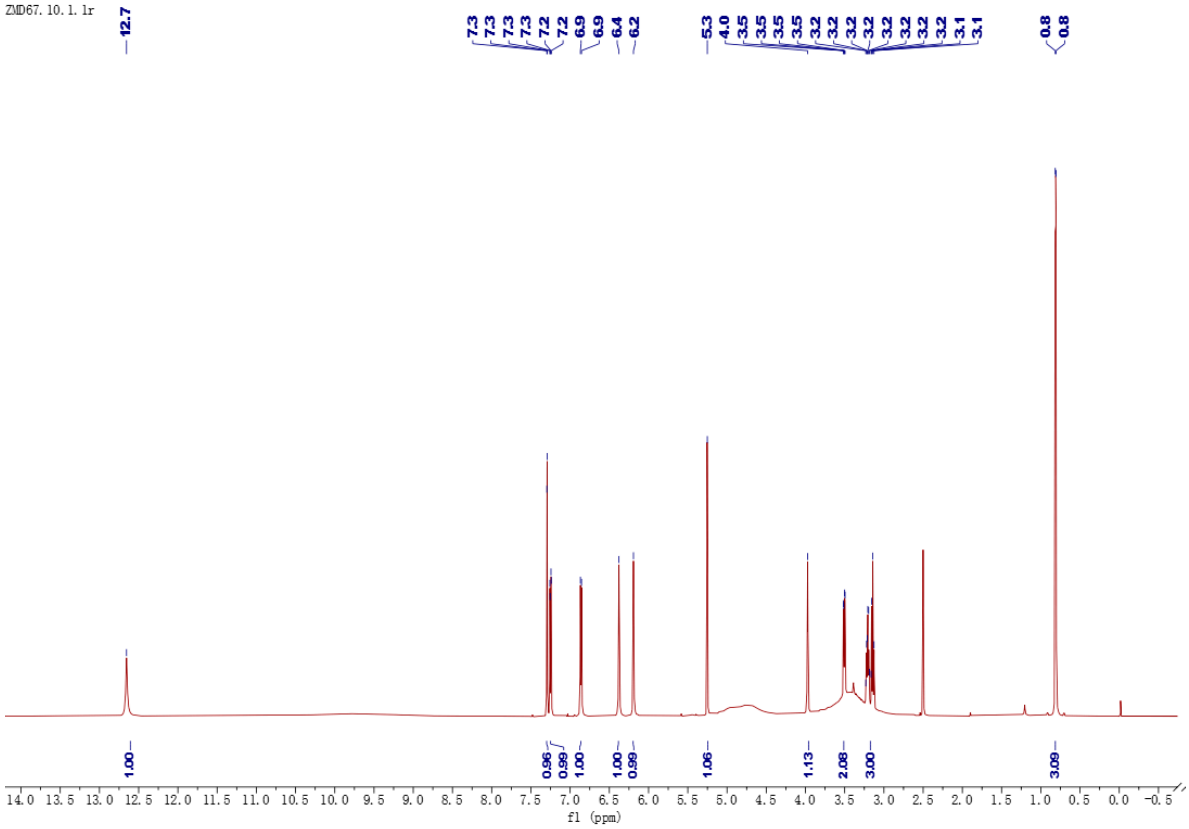

Figure S54: ^{1}H-NMR Spectrum of Compound **22**

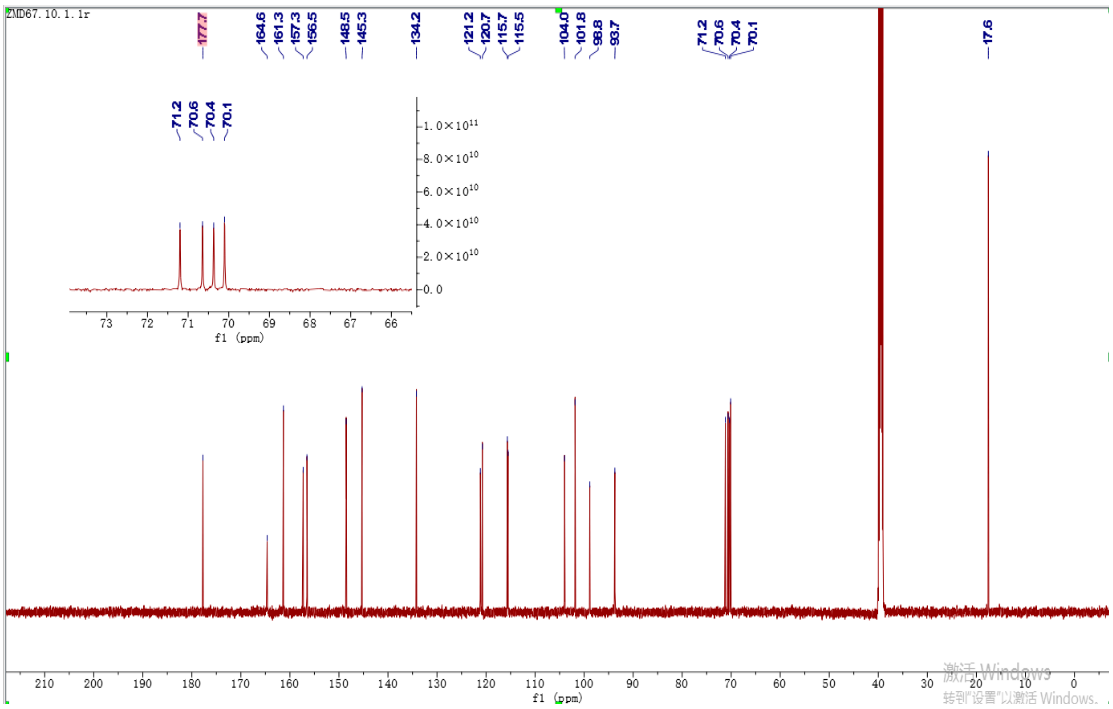

Figure S55: ^{13}C-NMR Spectrum of Compound **22**

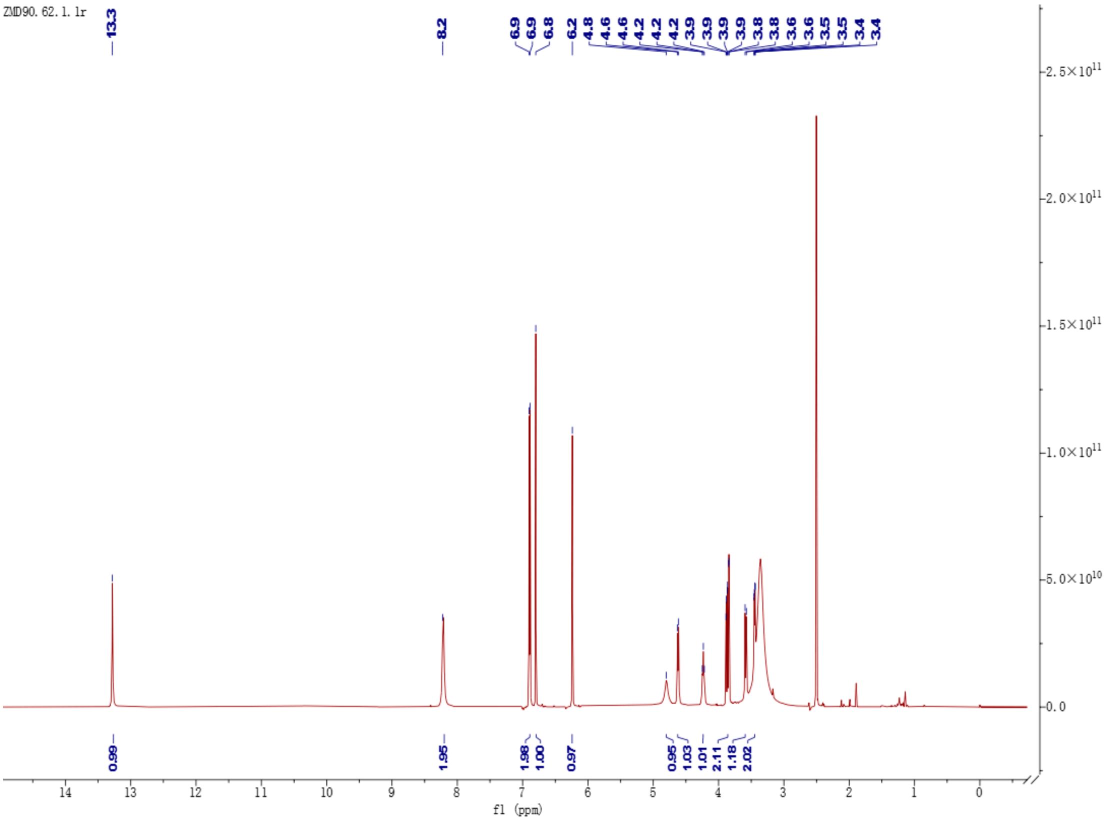

Figure S56: ^{1}H-NMR Spectrum of Compound **23**

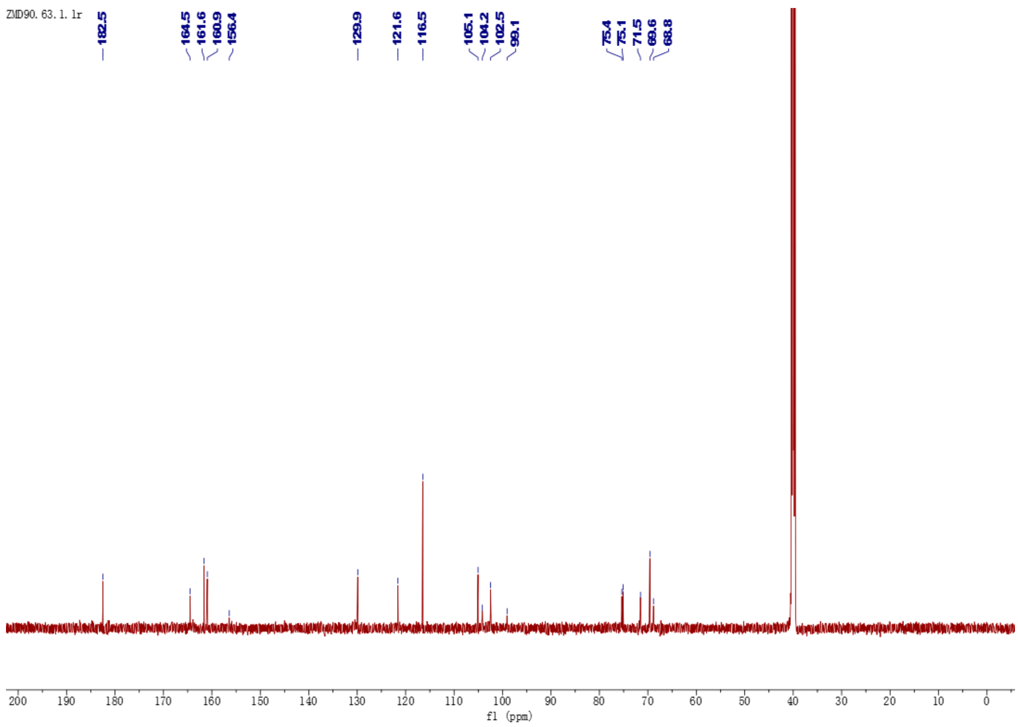

Figure S57: ^{13}C-NMR Spectrum of Compound **23**

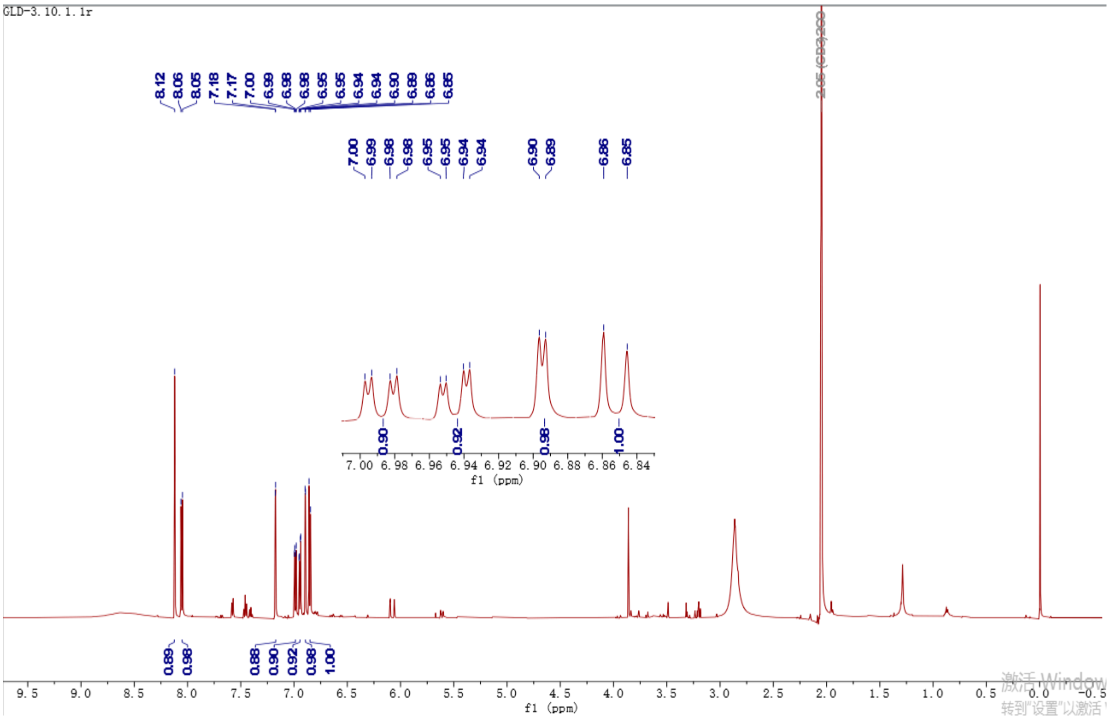

Figure S58: ^{1}H-NMR Spectrum of Compound **24**

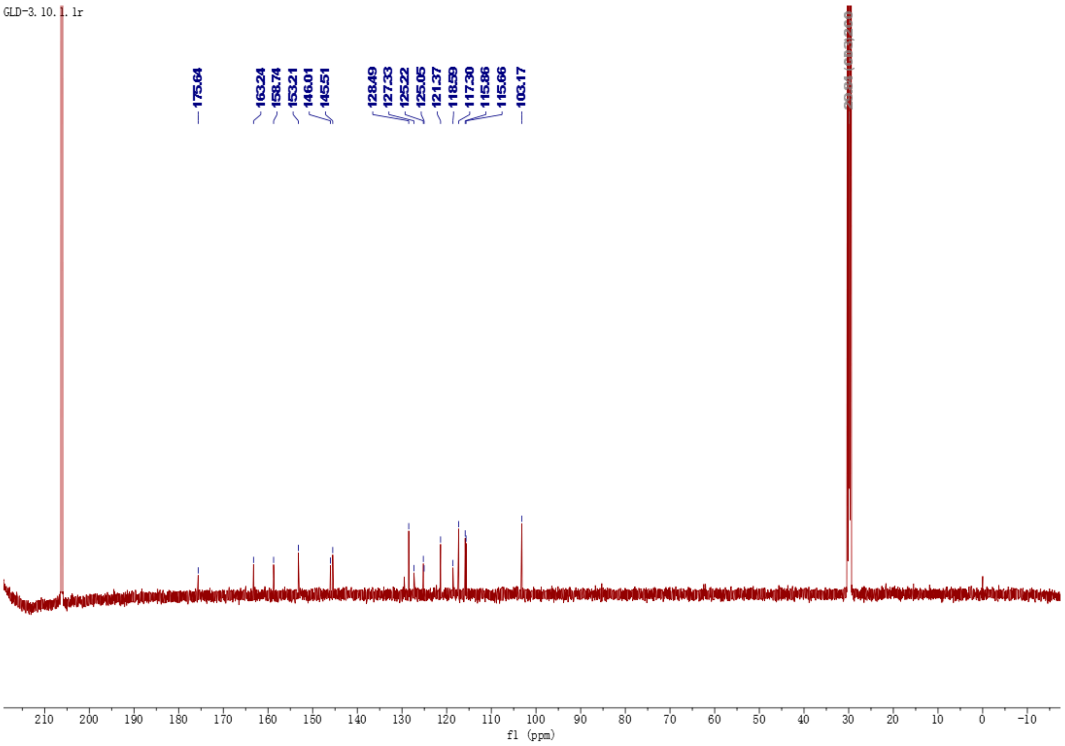

Figure S59: ^{13}C-NMR Spectrum of Compound **24**

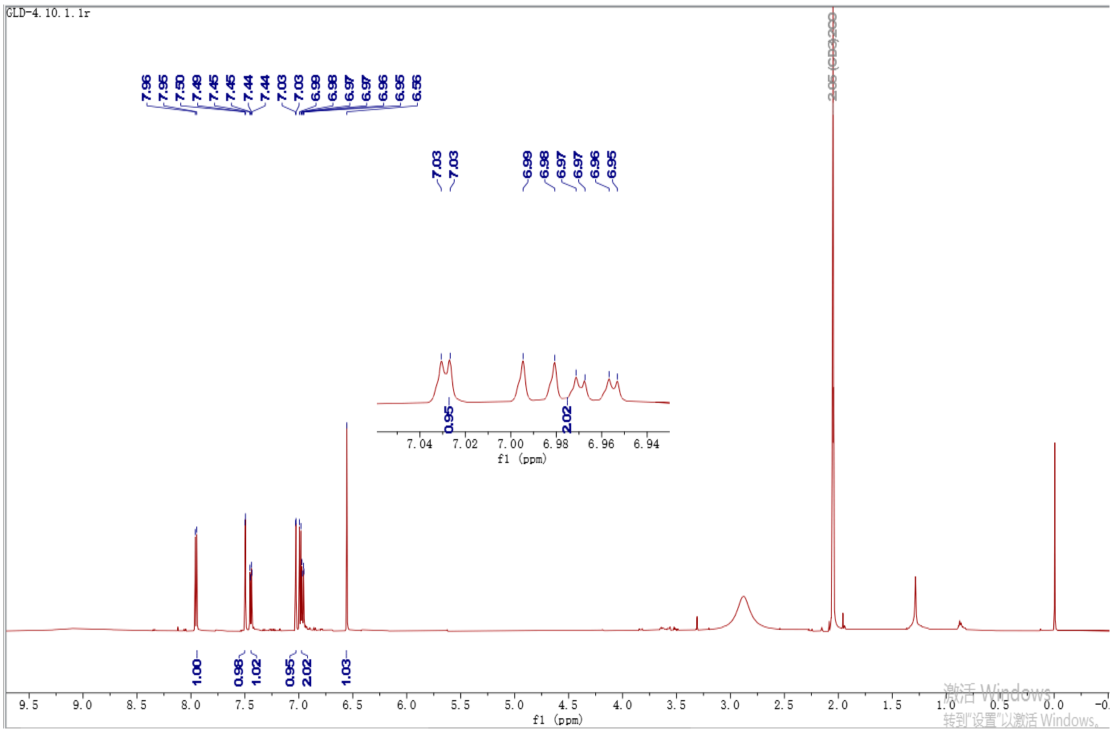

Figure S60: ^{1}H-NMR Spectrum of Compound **25**

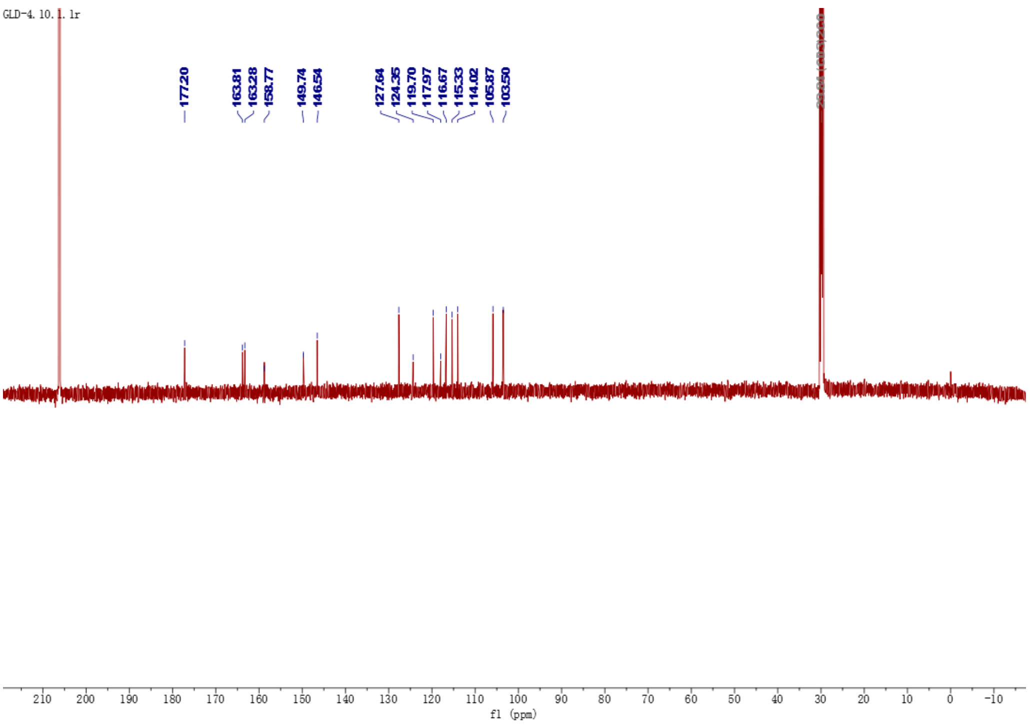

Figure S61: ^{13}C-NMR Spectrum of Compound **25**

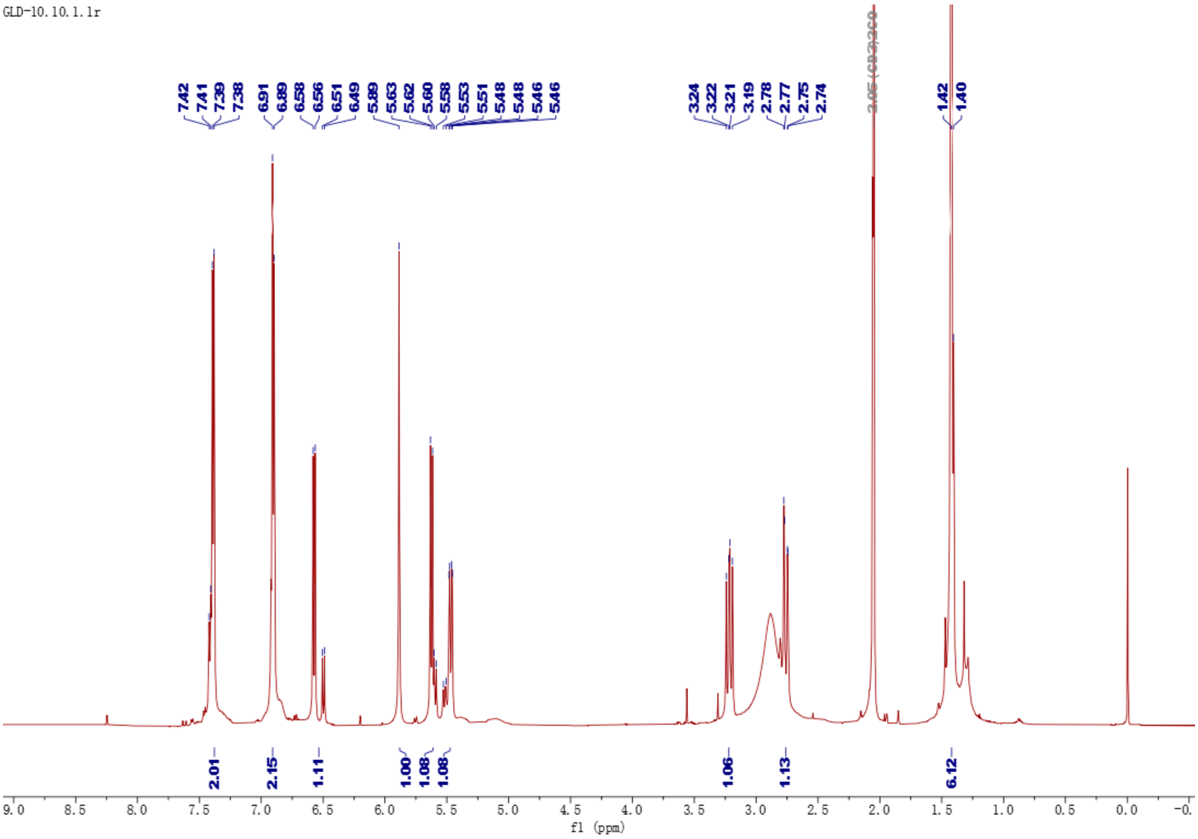

Figure S62: ^{1}H-NMR Spectrum of Compound **26**

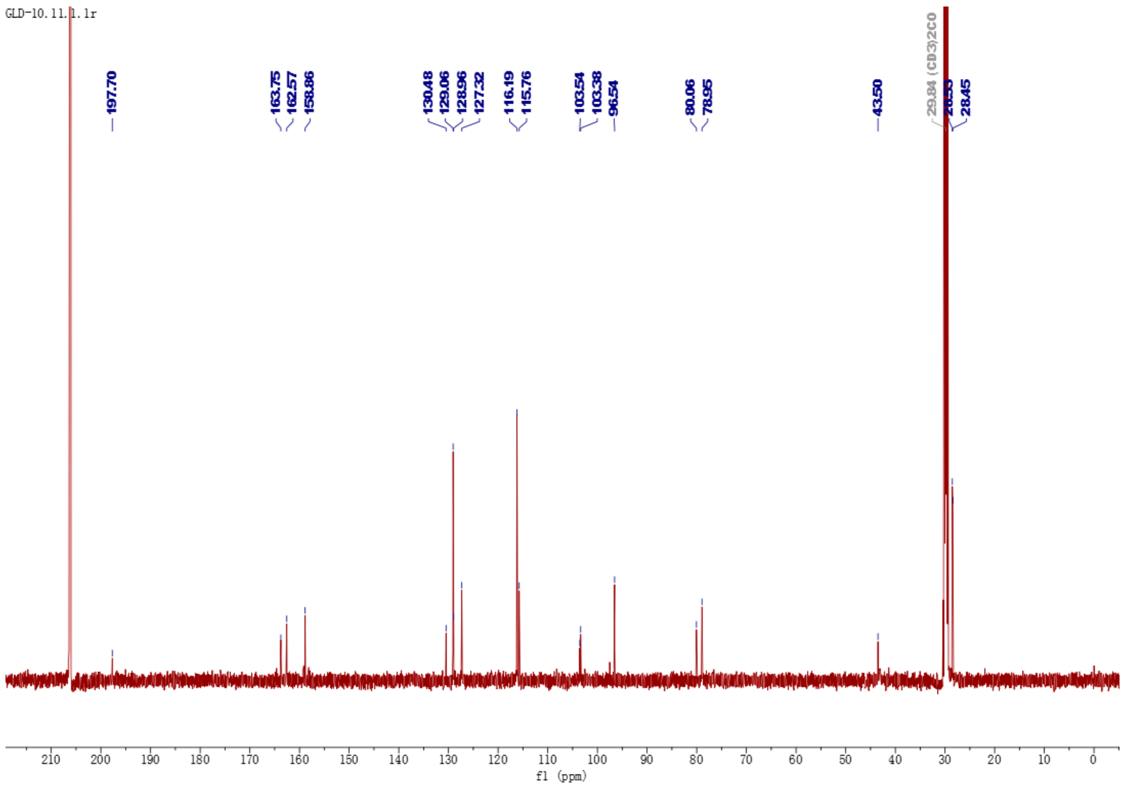

Figure S63: ^{13}C-NMR Spectrum of Compound **26**

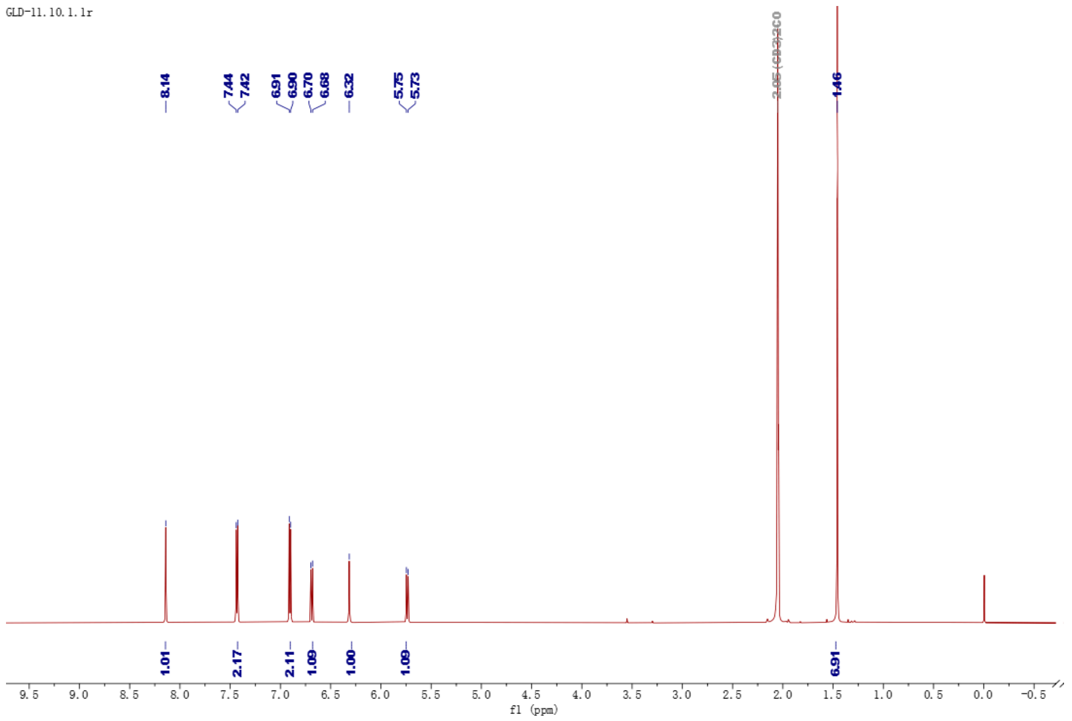

Figure S64: ^{1}H-NMR Spectrum of Compound **27**

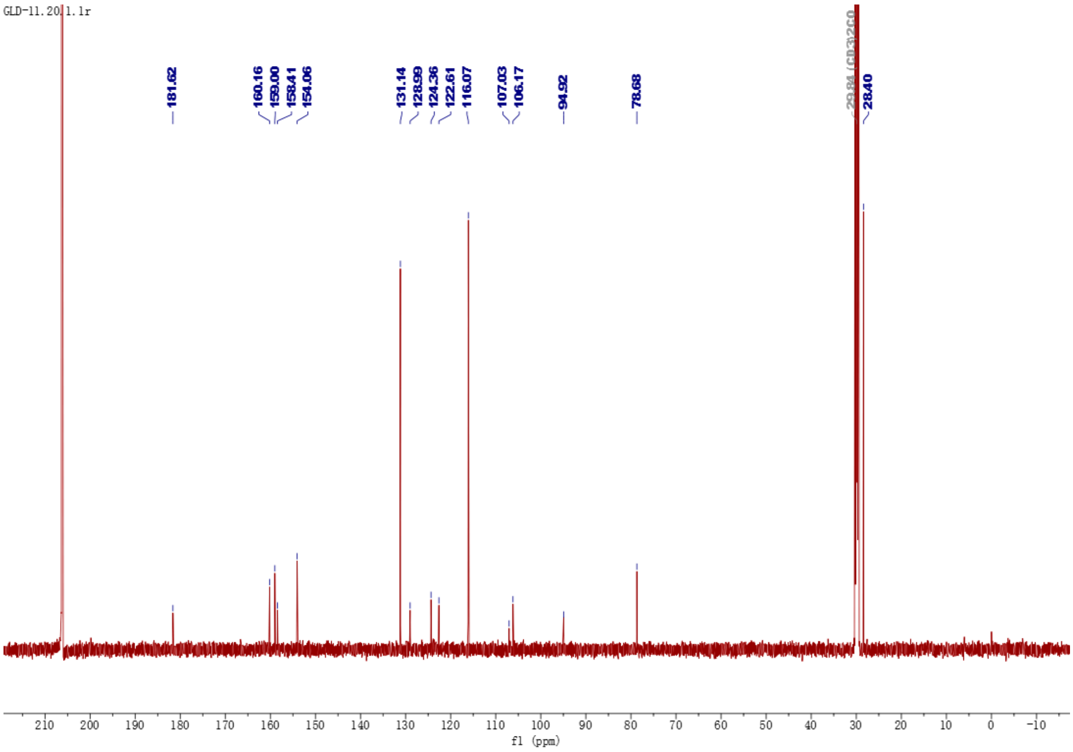

Figure S65: ^{13}C-NMR Spectrum of Compound **27**
